# Swedish Alzheimer’s disease variant perturbs activity of retrograde molecular motors and causes widespread derangement of axonal transport pathways

**DOI:** 10.1016/j.jbc.2024.107137

**Published:** 2024-03-05

**Authors:** Monica Feole, Victorio M. Pozo Devoto, Neda Dragišić, Cayetana Arnaiz, Julieta Bianchelli, Kateřina Texlová, Kristina Kovačovicova, Jan S. Novotny, Daniel Havas, Tomas L. Falzone, Gorazd B. Stokin

**Affiliations:** 1 Translational Ageing and Neuroscience Program, Centre for Translational Medicine, International Clinical Research Centre, St Anne’s University Hospital, Brno, Czech Republic; 2 Faculty of Medicine, Department of Biology, Masaryk University, Brno, Czech Republic; 3 School of Cardiovascular and Metabolic Medicine & Sciences, King's College London, London, UK; 4 Instituto de Investigación en Biomedicina de Buenos Aires (IBioBA-CONICET-MPSP), Partner Institute of the Max Planck Society, Buenos Aires, Argentina; 5 PsychoGenics, Paramus, New Jersey, USA; 6 Institute for Molecular and Translational Medicine, Faculty of Medicine and Dentistry, Palacký University Olomouc, Olomouc, Czech Republic; 7 Instituto de Biología Celular y Neurociencia IBCN (UBA-CONICET), Facultad de Medicina, Universidad de Buenos Aires, Buenos Aires, Argentina; 8 Division of Neurology, University Medical Centre, Ljubljana, Slovenia; 9 Department of Neurosciences, Mayo Clinic, Rochester, Minnesota, USA

**Keywords:** Alzheimer’s disease, axonal transport, familial pathogenic variants, amyloid precursor protein, dynactin-1, early endosomes, lysosomes

## Abstract

Experimental studies in flies, mice, and humans suggest a significant role of impaired axonal transport in the pathogenesis of Alzheimer’s disease (AD). The mechanisms underlying these impairments in axonal transport, however, remain poorly understood. Here we report that the Swedish familial AD mutation causes a standstill of the amyloid precursor protein (APP) in the axons at the expense of its reduced anterograde transport. The standstill reflects the perturbed directionality of the axonal transport of APP, which spends significantly more time traveling in the retrograde direction. This ineffective movement is accompanied by an enhanced association of dynactin-1 with APP, which suggests that reduced anterograde transport of APP is the result of enhanced activation of the retrograde molecular motor dynein by dynactin-1. The impact of the Swedish mutation on axonal transport is not limited to the APP vesicles since it also reverses the directionality of a subset of early endosomes, which become enlarged and aberrantly accumulate in distal locations. In addition, it also reduces the trafficking of lysosomes due to their less effective retrograde movement. Altogether, our experiments suggest a pivotal involvement of retrograde molecular motors and transport in the mechanisms underlying impaired axonal transport in AD and reveal significantly more widespread derangement of axonal transport pathways in the pathogenesis of AD.

The amyloid precursor protein (APP) is a type I integral membrane protein active at synapses (1) and best known for its role in the amyloid pathology and pathogenesis of AD (2). In fact, autosomal dominant variants of APP have long been identified to segregate with kindreds afflicted by AD (3). Although exceptionally rare, these familial AD (FAD) variants play an invaluable role in elucidating mechanisms underlying the pathogenesis of AD. For example, the APP KM670/671NL Swedish double variant (APP_swe_) promotes β-cleavage of APP at the N terminus of its amyloid-β peptide (Aβ) sequence (4). This cleavage enhances the formation of β-cleaved APP C-terminal fragments (β-CTFs), which are subject to γ-cleavage at the C terminus of the Aβ sequence and as a result, release an excess of Aβ (5, 6). Other FAD variants, such as the APP V717I London (APP_lon_), promote γ- rather than β-cleavage and also release an excess of Aβ^,^ (7, 8). Aberrant Aβ production spearheads the amyloid cascade hypothesis, which postulates that Aβs ignite and drive the pathogenesis of AD (9). Pathogenic FAD variants, however, also increase β-CTFs levels and enlarge early endosomes, which suggests that mechanisms of perturbed intracellular sorting and degradation are likewise at play in the pathogenesis of AD (10, 11, 12, 13).

In the axons, APP undergoes fast axonal transport (14) and proteolytic cleavage into β-CTFs and Aβ (15). Although interactions between the components of the APP motor assemblies remain to be further elucidated (16, 17), a number of studies report that APP vesicles move within the axons by highly processive molecular motors, the kinesin-1 in the anterograde, and the dynein–dynactin (DCTN1) complex in the retrograde direction (18, 19, 20, 21). Components of both anterograde and retrograde molecular motors undergo changes with aging (22, 23, 24, 25), the major risk factor of sporadic AD (26), and cause axonal transport impairments in a plethora of neurodegenerative disorders (27, 28, 29, 30). Some of these studies report associations between dynein dysfunction, bidirectional impairments in axonal transport, increased APP β-cleavage in the endosomal compartment, and aberrant cellular Aβ accumulation (23, 31). Studies in animal models and patients afflicted by FAD point to the role of axonal transport in the pathogenesis of AD and suggest that FAD variants perturb axonal transport (32, 33, 34, 35, 36). These studies have gained further support from cell culture experiments, which demonstrate that FAD variants reduce the proportion of anterogradely transported APP and that this phenotype can be reversed by blocking the β-cleavage site of APP (37). The changes in the transport properties of the cargoes and in the processivity of molecular motors underlying a putative transport disruption by FAD variants, however, remain unknown. We here rigorously characterize the effects of the Swedish FAD APP variant on axonal transport and provide an insight into the mechanisms and extent by which this variant impairs axonal transport. These findings are supplemented with the preliminary characterization of the effects of the APP_lon_ on the axonal transport. Here, presented work reveals that impairments elicited by APP_swe_ and likely also by other FAD variants involve retrograde transport machinery and cause significantly more widespread derangements of the axonal transport pathways than previously thought.

## Results

### Different pathogenic FAD variants impair bidirectional APP movement in human axons

To examine whether different pathogenic FAD variants of APP impair its axonal transport, we recreated previously reported experimental settings (37) using this time human neurons. In brief, human neural stem cells (hNSCs) were differentiated towards mature neurons for 38 DIV and then transfected with either WT APP (APP_wt_) or APP harboring Swedish (APP_swe_) or London (APP_lon_) FAD variant, all linked to GFP (Fig. S1*A*). The movies of GFP particle transport were acquired 2 days following transfection from the distal neuronal projections (Movies S1–S3). The axonal transport captured by the movies was studied using two independent and yet complimentary axonal transport analytical approaches: the net analysis of the axonal transport, which measures the overall changes in the distance moved by the cargos in the anterograde and retrograde direction within the axon, and the segmental analysis, which measures changes in the distance moved by the cargos in each segment of their trajectories.

In agreement with previous work (37), the net analysis of the axonal transport revealed a significantly reduced proportion of anterogradely transported APP_swe_ particles compared with APP_wt_ (Fig. S1*B*), whereas no significant changes in the average velocities were detected when comparing the three APP variants (Fig. S1*C*). To further investigate these confirmatory findings (37), we applied an enhanced method based on segmental analysis of the axonal transport. This analysis revealed significantly increased retrograde time in motion as well as in pause and reversion frequencies of both examined FAD APP variants compared with APP_wt_ (Fig. S1, *D*–*F*). These experiments thus not only corroborate and extend previous observations by showing that different FAD APP variants disrupt axonal transport (37) but specifically that FAD variants of APP impair its bidirectional movement.

### Swedish pathogenic variant causes a significant standstill of APP in the axons

Previous work has shown that the Swedish variant reduces anterograde axonal transport of APP in the primary cultures of mature mouse hippocampal neurons (37). This work, together with our observation that the Swedish variant perturbs bidirectional movement of APP more profoundly than the London variant, prompted us to investigate further this event by studying specifically the effects of the Swedish variant on the net APP transport in axons from hNSCs-derived neurons.

**Figure 1:**
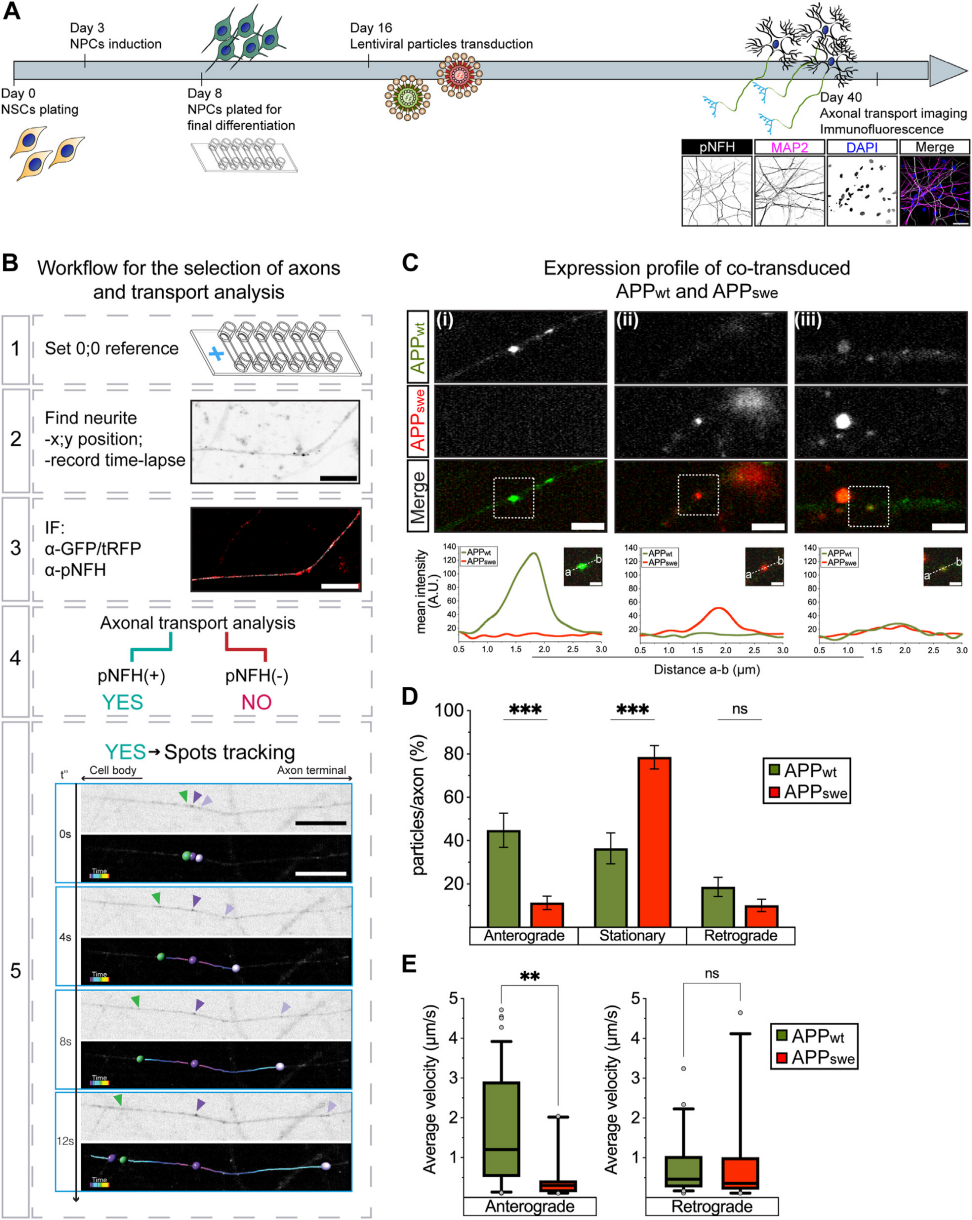
**Swedish FAD variant increases stationary APP particles by reducing anterograde APP transport.***A*, depiction of neuronal stem cell differentiation, lentiviral transductions, and time-points of axonal transport imaging followed by the immunofluorescence. Representative micrographs of neurons stained against pNFH, MAP2, and DAPI (*lower right*, scale bar represents 50 μm). *B*, experimental workflow for the selection of axonal processes from the neurons grown in ibidi μ-Slides VI used in the axonal transport analysis: (1) reference point setting, (2) localization of neurites for time-lapse recording, (3) post live-imaging immunofluorescence (IF) of neurites staining either against GFP (APP_wt_) or tRFP (APP_swe_) tag, and against pNFH, (4) inclusion of pNFH(+) and exclusion of pNFH(−) neurites from the axonal transport analysis, and (5) representative time frames of APP particles moving in the pNFH(+) axon terminal. Each frame consists of live (*above*) and processed (*below*) images *via* semi-automated tracking (scale bar represents 20 μm). *C*, micrographs representative of live imaged neurons transduced with APP_wt_ GFP and APP_swe_ tRFP (scale bar represents 5 μm). Relative intensity quantification of APP spots (last raw): representative case (i) where APP_wt_ GFP signal intensity is higher than APP_swe_ tRFP signal, representative case (ii) where APP_swe_ tRFP signal intensity is higher than APP_wt_ GFP signal, and representative case (iii) where APP_wt_ GFP and APP_swe_ tRFP both exhibit low signal intensities (outset images, scale bar represents 2 μm). *D*, proportions of APP_wt_ and APP_swe_ particles per axon moving in anterograde or retrograde direction or stationery (*n* = 15 axons from 3 biological replicates). *E*, average velocities of anterogradely or retrogradely moving APP_wt_ and APP_swe_ (*n* > 10 particles per treatment from three biological replicates). Data show mean intensities of particles over distances (a-b segments) (*C*), mean ± s.e.m. (*D*), or 10 to 90 percentile’s box-and-whiskers (*E*). Statistical comparisons were performed using 2-way ANOVA followed by Šídák's multiple comparisons test (*D*) and Mann-Whitney *U* test (E) (∗∗*p* <0.01, ∗∗∗*p* <0.001). APP, amyloid precursor protein; FAD, familial AD; tRFP, turbo red fluorescent protein.

To allow for simultaneous recording of both APP_wt_ and APP_swe_ in individual axons, hNSC-derived neurons were transduced with either APP_wt_ linked to GFP or APP_swe_ linked to turbo red fluorescent protein (tRFP) and further differentiated in ibidi multichannel devices for a total of 40 DIV (Fig. 1*A*). At this point, the movies of APP_wt_ and APP_swe_ particles were acquired from the same distal neuronal projections (Fig. 1*B*-box 1 and 2 and the cultures stained for either GFP or tRFP and a well-established axonal (pNFH) marker (38) (Fig. 1*B*-box 3). We used position retrieval to select only movies in which GFP or tRFP fluorescence overlapped with the pNFH marker for the analysis of axonal transport (Fig. 1*B*-box 4). APP movement was then measured within these axons using a semi-automated tracking algorithm (Fig. 1*B*-box 5).

When we assessed the quality of the movies of axons cotransduced with APP_wt_ and APP_swe_, we noted a sufficiently strong signal for the analysis of only either APP_wt_ -GFP (Fig. 1*C*(i)) or APP_swe_ -tRFP (Fig. 1*C*(ii)) but never of both in the same axons (Fig. 1*C*(iii)). Post-imaging–fixed cultures show divergent localization of the GFP and tRFP tags, which precluded simultaneous assessment of APP_wt_ and APP_swe_ movement within the same axons (Fig. S2*A*). To circumvent this pitfall, we first excluded the possible confounding effect of different tags by transducing neuronal cultures with either APP_wt_-GFP (Movie S4) or APP_wt_-tRFP (Fig. S2*B*; Movie S8) and demonstrating comparable net axonal transport readouts between APP_wt_ coupled to either GFP or tRFP (Fig. S2*C*). Then, we performed the net analysis of axonal transport in hNSCs-derived neurons transduced independently with either APP_wt_ -GFP or APP_swe_ -tRFP (Movies S4 and S5).

Measurements showed a significant reduction of anterogradely transported APP_swe_ compared with APP_wt_ (Fig. 1*D*). Strikingly, this reduction was accompanied by a significant increase in stationary APP_swe_ (app. 80%) compared with APP_wt_ (app. 40%) particles. Also, APP_swe_ exhibited a significant decrease in average anterograde velocity compared with APP_wt_ (Fig. 1*E*-*left*), but not in average retrograde velocity (Fig. 1*E*-*right*). To confirm that the observed impairments in the transport of APP_swe_ are exclusively axonal, we further validated our findings by analyzing axonal APP_wt_ and APP_swe_ transport in axonal compartment of the microfluidic chambers cultured with hNSCs-derived neurons (Fig. S3*A*) and confirmed a significant decrease in anterograde proportions of APP_swe_ compared to APP_wt_ as well as increase in stationary (Fig. S3*B*). In summary, by employing independent experimental paradigms designed to examine APP_wt_ and APP_swe_ transport exclusively in axons, we have demonstrated that the major “net” effect of the Swedish variant is a significant standstill of APP particles in the axons due to a reduced proportion and average velocity of anterogradely transported APP.

### Swedish pathogenic variant precludes APP from reaching its distal outposts in the axon

**Figure 2:**
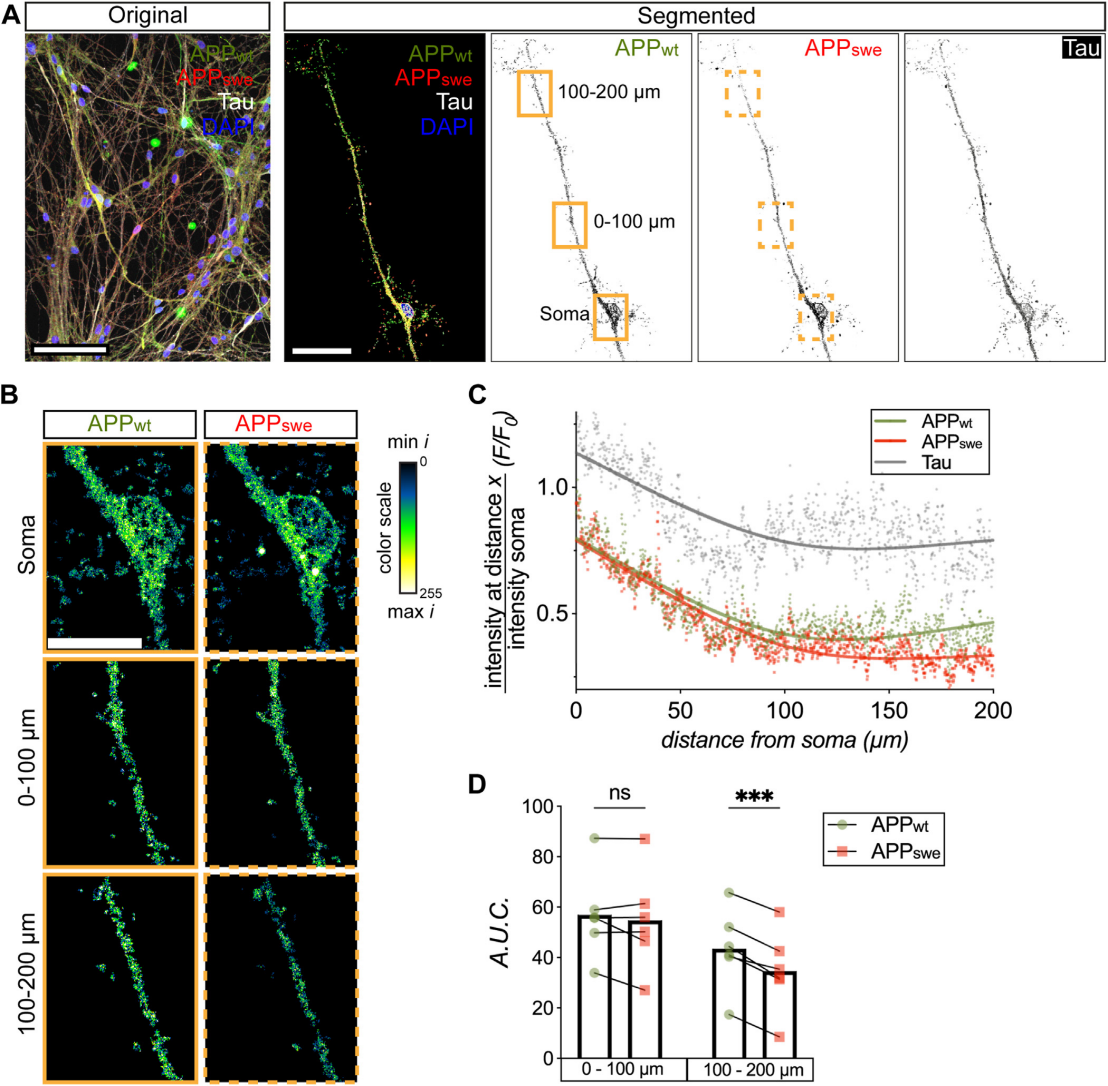
**Swedish FAD variant perturbs distribution of APP along the axons.***A*, representative image of a neuronal culture cotransduced with APP_wt_ GFP and APP_swe_ tRFP followed by staining against GFP, tRFP, tau, and DAPI (*left*, scale bar represents 100 μm). Zoom-in and segmentation of a neuron-expressing APP_wt_, APP_swe_, and tau. *Yellow* boxes indicate the region of interest detailed in (*B*) (*right*, scale bar represents 50 μm). *B*, high magnification pseudo-color images of the soma and the axon at <100 μm and >100 μm from the soma (scale bar represents 20 μm; color scale bar = min intensity 0 – max intensity 255). *C*, graph showing the intensity (*f*) of APP_wt_, APP_swe_, and Tau signals along each point of the axon (0–200 μm) relativized to their respective mean intensity in the soma (*f*_*0*_). For each protein, the dots represent the mean intensity values at distance *x* from the soma, fitted and smoothed by a continuous line (*n* = 20 neurons from six biological replicates). *D*, quantification of the total intensity (A.U.C.: area under the curve) of APP_wt_ and APP_swe_ along the axon from 0 to 100 μm and 100 to 200 μm from the soma (*n* = 20 neurons from six biological replicates). Data are shown as mean intensity values (*C*) and A.U.C. values with mean bar (*D*). Statistical comparison was performed using 2-way ANOVA (matched by biological replica) followed by Šídák's multiple comparisons test (*D*) (∗∗∗*p* < 0.001). APP, amyloid precursor protein; FAD, familial AD; tRFP, turbo red fluorescent protein.

The observed stagnation of APP within axons produced by the Swedish variant prompted us to investigate whether APP_swe_ can reach its physiological locations across the entire axon at all. To this end, we examined the axonal distribution of APP in two parallel experimental settings: (i) neurons cotransduced with APP_wt_-GFP as well as with APP_swe_-tRFP and (ii) neurons cotransduced with APP_wt_-GFP as well as with APP_wt_-tRFP (control experiment) (Figs. 2*A* and S4*A*). These neurons were then stained for GFP and tRFP as well as for tau as an internal control and the distribution of different fluorescent signals within the same axons in both experimental settings was assessed (Figs. 2B and S4*B*). The assessment of the distribution of APP_wt_-GFP and APP_swe_-tRFP in the same axons revealed that the axonal APP_swe_ signal progressively declines further away from the soma (Fig. 2*C*). In contrast, the distribution of APP_wt_ coupled to either GFP or tRFP remained identical across the entire axons in the control experiment, which demonstrated that the use of different fluorescent tags does not affect the localization or the detection of APP within neurons and in particular within axons (Fig. S4, *C* and *D*).

To further test this observation, we compared the total normalized intensities of APP_wt_ *versus* APP_swe_ signal in the proximal (0–100 μm) and distal axonal segments (100–200 μm) (Fig. 2*D*). Within their first 100 μm from the soma, axons exhibited comparable total intensities of the APP_wt_ GFP and APP_swe_ tRFP signal. In stark contrast, at distances greater than 100 μm, axons showed a significant reduction in the total intensities of APP_swe_-tRFP when compared with APP_wt_-GFP. These experiments indicate that the Swedish variant precludes APP from reaching its physiological location in the distal outposts of the axons.

### Swedish pathogenic variant perturbs coordinated activities between molecular motors involved in bidirectional APP movement

**Figure 3:**
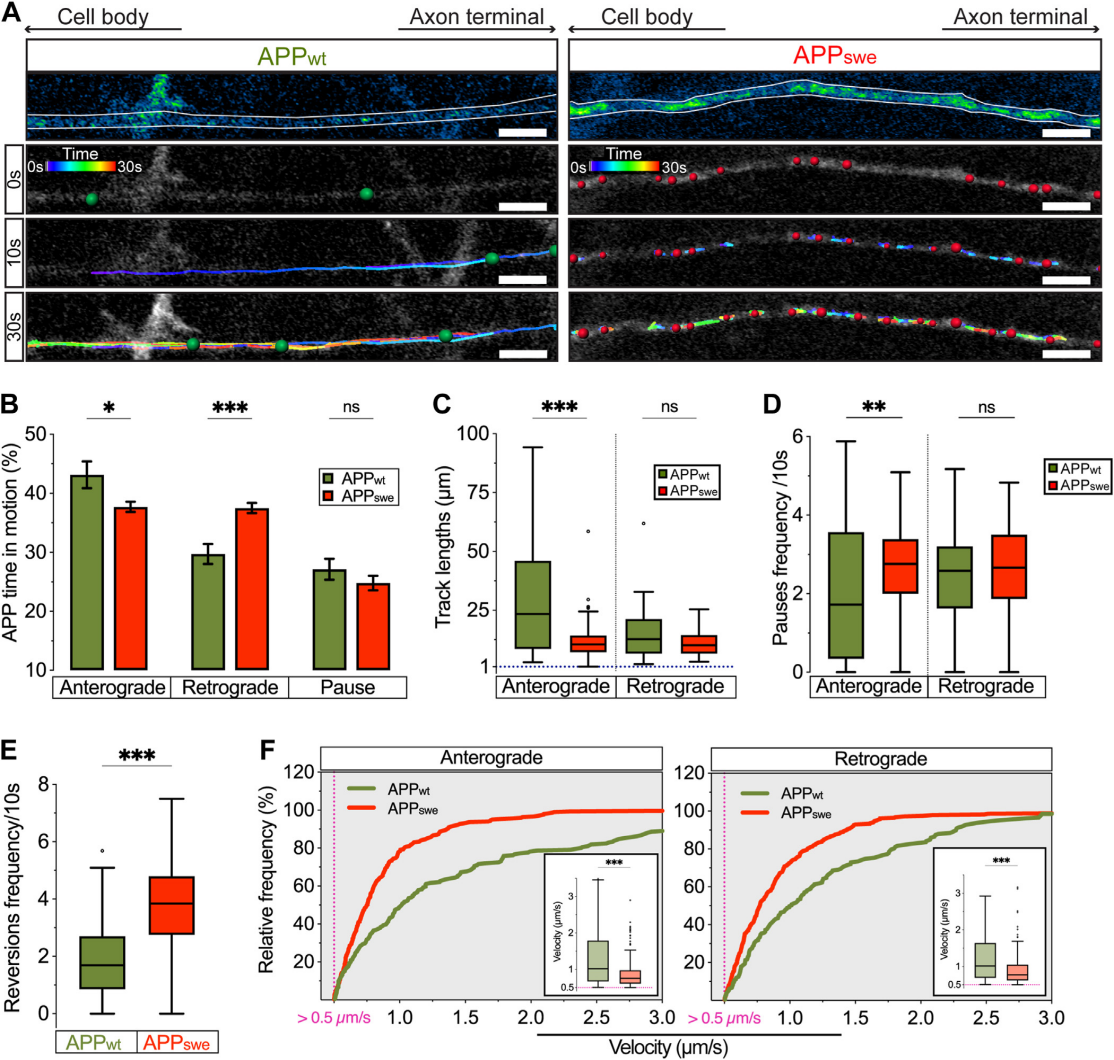
**Swedish variant enhances retrograde time in motion and reduces the velocity of APP transport.***A*, representative micrographs of the axonal transport of APP_wt_ (*left*) and APP_swe_ (*right*) showing the overall movement of APP at different time points (0, 10, and 30 s; scale bar represents 10 μm). *B*, percent real-time motion, anterograde or retrograde, and pausing of APP_wt_ and APP_swe_ particles (*n* > 150 particles from three biological replicates). *C*, quantification of track lengths (distances) reached by either anterogradely or retrogradely transported APP_wt_ and APP_swe_ particles (*n* > 45 particles from three biological replicates). *D*, 10 s pauses frequency of anterogradely and retrogradely moving APP_wt_ and APP_swe_ particles (*n* > 45 particles from three biological replicates). *E*, 10 s reversion frequencies of APP_wt_ and APP_swe_ particles (*n* > 150 particles from three biological replicates). *F*, relative frequency of APP_wt_ and APP_swe_ segmental velocities, moving in anterograde *(left*) and retrograde (*right*) direction faster than 0.5 μm/s. Subgraphs show segmental velocities distribution as box-and-whiskers (*n* > 400 segments per population from three biological replicates). Data are represented as mean ± s.e.m. (*B*), Tukey’s box-and-whisker plot (*C*, *D*, *E*, and subgraphs *F*), and cumulative frequency distributions (*F*). Statistical comparisons were performed using 2-way ANOVA followed by Šídák's multiple comparisons test (*B*) and Mann-Whitney *U* test (C-F) (∗*p* < 0.05, ∗∗*p* < 0.01, ∗∗∗*p* < 0.001). APP, amyloid precursor protein.

To this point, our experiments indicate that the Swedish variant significantly halts axonal APP transport, which then fails to reach its distal outposts in the axons due to an overall reduction in its anterograde transport. To gain more detailed information about the changes in the axonal transport produced by the Swedish variant, we performed a segmental analysis of the axonal APP transport (Fig. 3*A*; Movies S6 and S7).

Complimentary to the findings of the net analysis, the segmental analysis revealed significant changes of both anterograde and retrograde particles in motion in APP_swe_ compared with APP_wt_ (Fig. 3*B*). More specifically, this analysis identified a switch in the percentage of APP particles undergoing anterograde and retrograde movement with significantly more particles moving retrogradely and less anterogradely in APP_swe_ compared with APP_wt_. In contrast to the increased percentage of APP_swe_ particles in retrograde motion, the shorter track lengths and increased pause frequencies were exhibited only by the APP_swe_ particles moving in the anterograde direction (Fig. 3, *C* and *D*). These findings were accompanied by a significant increase in the overall reversion frequency (Fig. 3*E*) and significant decrease in anterograde (app. 0.5 μm/s slower) as well as retrograde (app. 0.3 μm/s slower) segmental velocities of the APP_swe_ compared with the APP_wt_ particles (Fig. 3*F*). The segmental analysis shows that the impaired anterograde APP transport by the Swedish variant is the consequence of abnormal retrograde movement. The significant standstill of APP particles by the Swedish variant is therefore the result of an overall slower and bidirectionally inefficient movement of APP_swe_ in comparison with APP_wt_. Collectively, these results suggest that the Swedish variant perturbs fine-tuned coordinated activities between the molecular motors involved in the bidirectional movement of APP in axons.

### Swedish pathogenic variant increases association of dynactin-1 with APP

**Figure 4:**
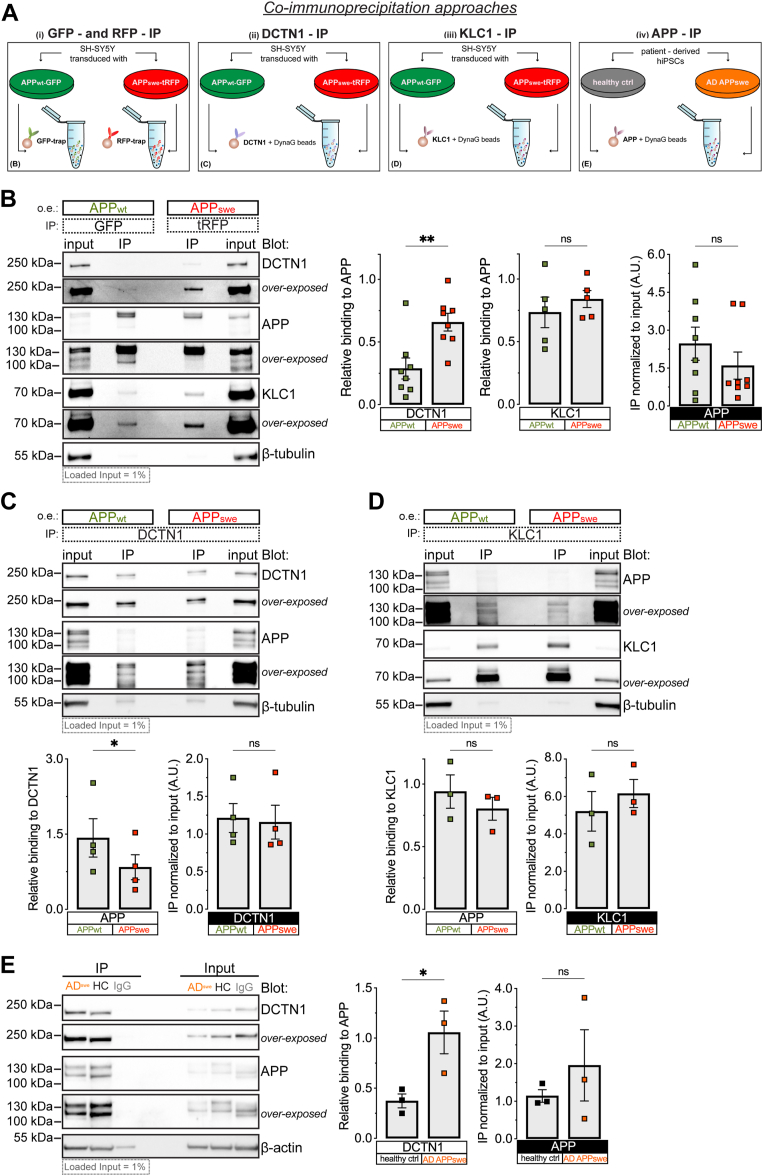
**Increased association of dynactin-1, but not of KLC1, with the APP by the Swedish FAD variant.***A*, schematic representation of co-immunoprecipitation approaches used in this study to evaluate in either differentiated SH-SY5Y cells or hiPSCs-derived from control and AD APP_swe_ patients, the interactions between APP, and anterograde and retrograde motor machinery: (i) GFP- and RFP-IP; (ii) DCTN1-IP; (iii) KLC1-IP; (iv) APP-IP. *B*, representative immunoblots of dynactin1 (DCTN1), APP, KLC1, and βIII-tubulin (loading control) following GFP or tRFP immunoprecipitations (IPs) from cell lysates of differentiated SH-SY5Y cells transduced with either APP_wt_ or APP_swe_. Co-IP ratios expressed as DCTN1 or KLC1 intensity relative to either APP_wt_ or APP_swe_ transduced samples (*left*, DCTN1 co-IPs *n* = 8 biological replicates; *middle*, KLC1 co-IPs, *n* = 6 biological replicates, entire individual blots are shown in Fig. S5*A*). IP efficiency of APP levels normalized by its corresponding input (10 μg of proteins per lane) from either APP_wt_ or APP_swe_ transduced cell cultures (right, *n* = 8 biological replicates). *C*, representative immunoblots of DCTN1, APP, and βIII-tubulin (loading control) following DCTN1 IPs performed with total protein lysates from either APP_wt_ or APP_swe_ transduced SH-SY5Y. Co-IP ratios expressed as APP intensity relative to DCTN1 in APP_wt_*versus* APP_swe_ transduced cell cultures (*left*, *n* = 4 biological replicates, entire individual blots are shown in Fig. S7*A*). IP efficiency of DCTN1 levels normalized by its corresponding input (10 μg of proteins per lane) from either APP_wt_ or APP_swe_ transduced cell cultures (right, *n* = 4 biological replicates). *D*, representative immunoblots of APP, KLC1, and βIII-tubulin (loading control) of samples obtained from KLC1 IPs performed with total protein lysates from cell cultures transduced with either APP_wt_ or APP_swe_. Co-IPs ratios expressed as APP intensity relative to KLC1 in APP_wt_*versus* APP_swe_ transduced cell cultures (right, *n* = 3 biological replicates, entire individual blots are shown in Fig. S7*C*). IP efficiency of KLC1 levels normalized by its corresponding input (10 μg of proteins per lane) from either APP_wt_ or APP_swe_ transduced cell cultures (left, *n* = 3 biological replicates). *E*, representative immunoblots of DCTN1, APP, and β-actin (loading control) in total protein lysates of human iPSCs-derived neural progenitors from either healthy controls (HC) or AD APP_swe_ (AD^swe^) cell lines. Co-IP ratios expressed as DCTN1 intensity relative to APP in APP_wt_, APP_swe_, or IgG isotype control samples (right, *n* = 3 biological replicates, entire individual blots are shown in Fig. S8*A*). IP efficiency of APP levels normalized by its corresponding total lysate (TL, 10 μg of proteins per lane) from APP_wt_, APP_swe_, or IgG isotype controls (left, *n* = 3 biological replicates). Densitometries are shown as mean ± s.e.m. Statistical comparisons were performed using an unpaired *t* test (*B*–*E*) (∗*p* < 0.05, ∗∗*p* < 0.01). AD, Alzheimer’s disease; APP, amyloid precursor protein; FAD, familial AD; hiPSC, human induced pluripotent stem cell; IP, immunoprecipitation; KLC, kinesin light chain; tRFP, turbo red fluorescent protein.

The observed bidirectional impairment of axonal APP movement suggests perturbed coordination of activities between anterograde and retrograde molecular motors. To test for changes in the association between molecular motors and APP, we conducted a series of immunoprecipitation (IP) experiments using lysates from 20 DIV differentiated SH-SY5Y cells transduced either with APP_wt_ or APP_swe_ coupled to different combinations of tags as well as from human neural progenitors cells (hNPCs) differentiated from human induced pluripotent stem cells (hiPSCs) reprogrammed from a healthy subject and a patient affected by FAD carrying the Swedish variant (39) (Figs. 4A and S6*A*).

In the first set of experiments, SH-SY5Y cell lysates were IP-ed with either GFP or tRFP, which tagged APP_wt_ and APP_swe_, respectively, and the blots probed for APP, kinesin light chain 1 (KLC1), or dynactin-1 (DCTN1) and βIII-tubulin as the loading control (Figs. 4, *A*(i) and *B* and S5*A*).

The average ratios of co-IP-ed DCTN1, but not KLC1, were significantly increased in APP_swe_ compared with APP_wt_ cell lysates (Fig. 4*B*-*left and middle graph*). In support of these changes, there were no differences in the amounts of IP-ed *versus* total APP between the lysates prepared from cells transduced with either APP_wt_ or APP_swe_ as well as in the total amounts of APP, DCTN1, and KLC1 compared with the loading control (Fig. 4*B*-*right graph*; Fig. S5*B*). These results indicate that the observed increased amount of DCTN1 associated with APP is the consequence of the Swedish variant considering the same membranes reblotted for either GFP or tRFP recognized exclusively overexpressed APP, while IPs of nontransduced cultures showed no binding with antibodies against GFP or tRFP (Fig. S5, *C*–*E*).

To rule out the confounding effect of different tags on the association between DCTN1 and APP, we performed a second set of experiments where APP_wt_ and APP_swe_ were both tagged with tRFP and both IP-ed against tRFP (Fig. S6, *A* and *B*). Also in this experimental setting, the average ratios of co-IP-ed DCTN1, but not of KLC1, compared with IP-ed APP showed a significant increase in APP_swe_ compared with APP_wt_-transduced cell lysates (Fig. S6*C* - *left*). We did not observe any differences in the amounts of IP-ed *versus* total APP between lysates prepared from SH-SY5Y cells transduced with APP_wt_ and APP_swe_ as well as in the amounts of APP, DCTN1, and KLC1 normalized to the loading control (Fig. S6, *C*-*right* and *D*). This control experiment indicates that the increased amount of DCTN1 associated with APP is not the result of the confounding effect of IP-ing APP using different tags but rather the consequence of the Swedish variant.

In the third set of experiments, we examined further the observed increased association of DCTN1 with APP by IP-ing SH-SY5Y cell lysates with DCTN1 or KLC1 and probing the blots for APP, DCTN1, KLC1, and βIII-tubulin (Figs. 4*A*(ii), *C* and *D* and S7, *A* and *C*). The average ratios of co-IP-ed APP with DCTN1, but not with KLC1, showed a significant decrease in co-IP-ed APP_swe_ compared with APP_wt_ (Fig. 4, *C* and *D* – *left graphs*). We found no differences in the amounts of IP-ed *versus* total DCTN1 or KLC1 between the lysates prepared from SH-SY5Y cells transduced with APP_wt_ and APP_swe_ (Fig. 4, *C* and *D* – *right graphs*), indicating more DCTN1 available to bind APP_swe_. No differences were found in the total amounts of APP, DCTN1, and KLC1 compared with the loading control (Fig. S7, *B* and *D*). These results again indicate that the Swedish variant underlies the observed increased number of DCTN1 molecules associated with APP.

To test whether the observed change in the association between DCTN1 and APP_swe_ can be replicated in a clinically relevant experimental model of AD, we next examined hiPSC generated from fibroblasts of a healthy donor and a patient with FAD carrying the Swedish variant. In this fourth set of experiments, the lysates prepared from hNPCs differentiated from hiPSCs were IP-ed using either an antibody against APP or against an IgG, and the blots probed for APP, DCTN1, and β-actin as the loading control (Figs 4*A*(iv) and *E* and S8*A*). The average ratios of DCTN1 co-IP-ed with APP were significantly increased in hiPSCs-derived hNPCs differentiated from the FAD patient compared with those from a healthy subject (Fig. 4*E* – *left graph*). There were no differences in the amount of the IP-ed *versus* total APP between the cell lysates and also, total amounts of APP and DCTN1 normalized for their loading control did not change between the healthy subject and the AD patient (Figs. 4*E* – *right graph*; S8*B*). These results point to the endogenously expressed Swedish variant as responsible for the increased association of DCTN1 with APP.

To preliminarily investigate whether the Swedish variant increases the association of DCTN1 with APP by modulating the β-cleavage site of APP, we treated SH-SY5Y cells with either DMSO or with 10 or 40 μm concentration of a β-site APP-cleaving enzyme inhibitor (40), IP-ed lysates against tRFP, and probed the blots for APP, DCTN1, and β-actin as the loading control (Fig. S9, *A* and *B*). There were no differences in the total amounts of APP and DCTN1 normalized to the loading control as well as of IP-ed APP and co-IP-ed DCTN1 compared with their inputs between any of the treatments (Fig. S9, *C* and *D*). These preliminary results suggest that the observed increased association of DCTN1 with APP occurs independently from the modulation of the β-cleavage site by the Swedish variant.

Collectively, these experiments indicate that transduced or endogenously expressed APP carrying the Swedish variant invariably increases the association of DCTN1 with APP. Considering DCTN1 activates the retrograde molecular motor dynein (21), our experiments argue for increased activation of the dynein–dynactin complex and thus of the retrograde transport machinery by the Swedish variant, which occurs independently from its impact on the β-cleavage of the APP.

### Significant colocalization of dynactin-1 with APP in a mouse model of AD

**Figure 5:**
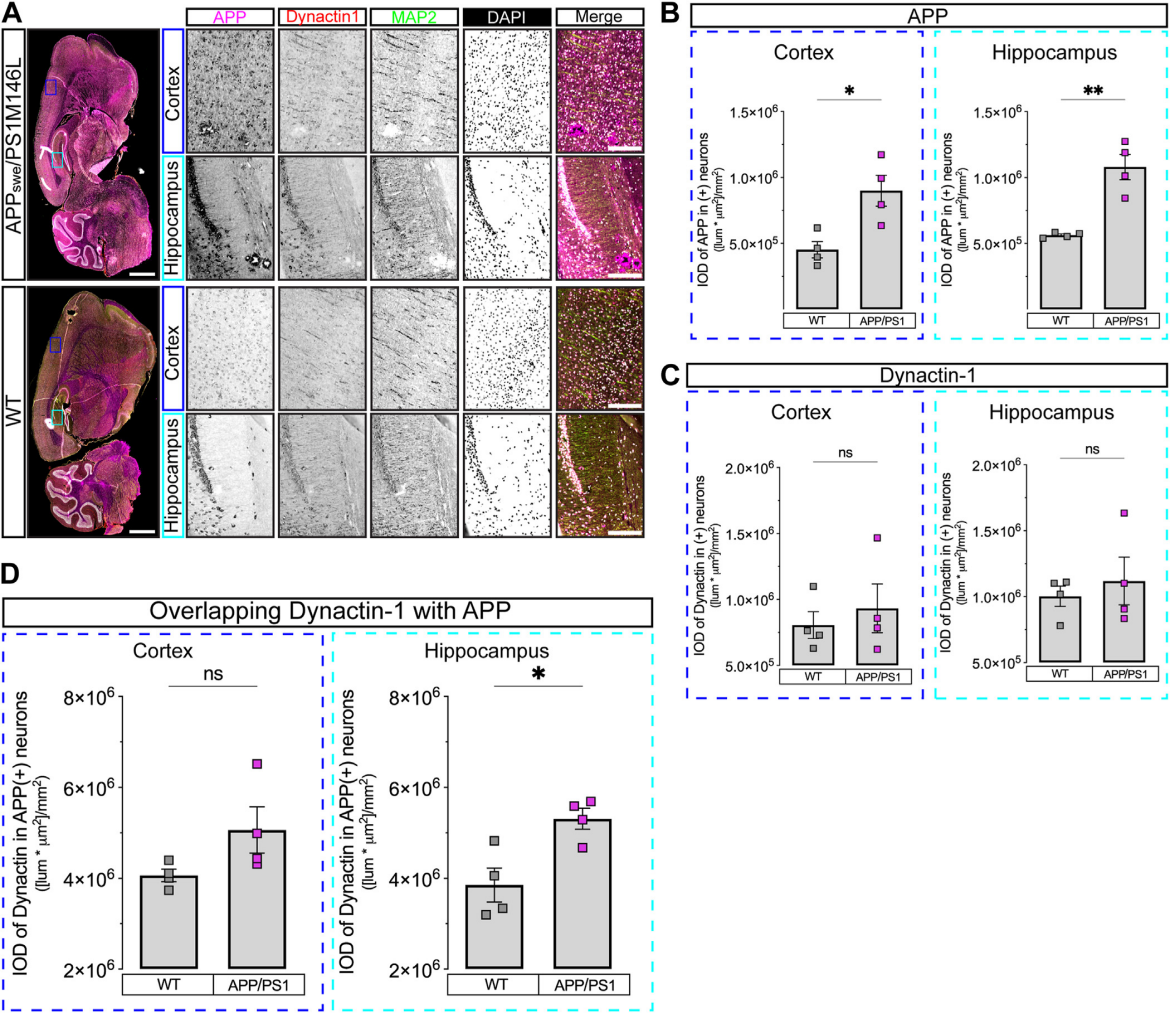
**Increased overlap between DCTN1 and APP in a mouse model of AD.***A*, representative full brain images from APP_swe_/PSEN1 M146L AD mouse model and WT stained for APP (*magenta*), DCTN1 (*red*), MAP2 (*green*), and DAPI (*white*) (scale bar represents 15 cm). Pictures show sagittal sections with highlighted ROIs in the cortex (*blue*) and hippocampus (*cyan*), which are shown on the side of the full brain micrographs as zoomed images (scale bar represents 2 cm). *B*, quantification of the APP intensities in MAP2(+) neurons measured in both cortex and hippocampus as normalized IODs (*n* = 4 brains from 10-month-old mice). *C*, quantification of the DCTN1 intensities in MAP2(+) neurons measured in both cortex and hippocampus as normalized IODs (*n* = 4 brains from 10-month-old mice). *D*, quantification of the DCTN1 intensities overlapping with APP intensities in MAP2(+) neurons measured in both cortex and hippocampus as normalized IODs ratios (*n* = 4 brains from 10-month-old mice). Data are shown as mean ± s.e.m. with individual values. Statistical comparisons were performed with the Mann-Whitney *U* test (B-D) (∗*p* < 0.05, ∗∗*p* < 0.01). AD, Alzheimer’s disease; APP, amyloid precursor protein.

To test whether the increased association of DCTN1 with APP in human neurons can be observed also in a pathophysiological setting relevant to AD, we examined transgenic mice expressing APP carrying the Swedish variant as well as Presenilin 1 (PSEN1) carrying the M146V variant, a *bona fide* model of AD (41). Brains harvested from 10-month-old age-matched WT and transgenic littermates were fixed, cut sagittally, and then stained against APP, DCTN1, MAP2 as an internal control and DAPI and the images of the brain sections acquired using a slide scanner (Fig. 5*A*).

The analysis of the APP staining in whole brain sections reiterated the previously reported increased integrated optical density (IOD) of APP signal in the cortex and in particular in the hippocampus of transgenic *versus* WT mice (Fig. 5*B*) (41). Transgenic but not WT mice also exhibited identifiable APP immunoreactive plaques in both brain regions examined (Fig. 5*A* – *close up – cortex* (blue), *hippocampus* (cyan)). These findings were not accompanied by any changes in the average IODs of the DCTN1 signal in the cortex or the hippocampus between WT and transgenic mice. APP immunoreactive plaques were also devoid of DCTN1 immunoreactivity (Fig. 5*C*). However, when we compared IODs of DCTN1 and APP signals, we observed significantly increased overlap between DCTN1 and APP in the hippocampus, but not in the cortex of the AD compared with the WT mouse model (Fig. 5*D*). These experiments indicate that APP overexpression in the setting of an AD mouse model results in significant colocalization between DCTN1 and APP in brain regions selectively impaired early in AD compared with WT mice. Although in agreement with the biochemically observed enhanced association between DCTN1 and APP, these experiments do not provide direct evidence further corroborating changes in their relationship.

### Swedish pathogenic variant promotes reversal of the axonal transport of the early endosomes

Several studies have reported that dynein-dynactin molecular machinery mediates the retrograde movement of a plethora of axonal organelles (42, 43, 44, 45). Considering that APP and its fragments populate several of these dynein-DCTN1 retrogradely driven organelles (17, 46, 47), the damage elicited by the Swedish variant is likely not limited to impaired coordination between the molecular motors involved in the movement of APP in one specific axonal compartment but rather encompasses several axonal compartments harboring APP with a significantly more widespread derangement of the transport pathways.

**Figure 6:**
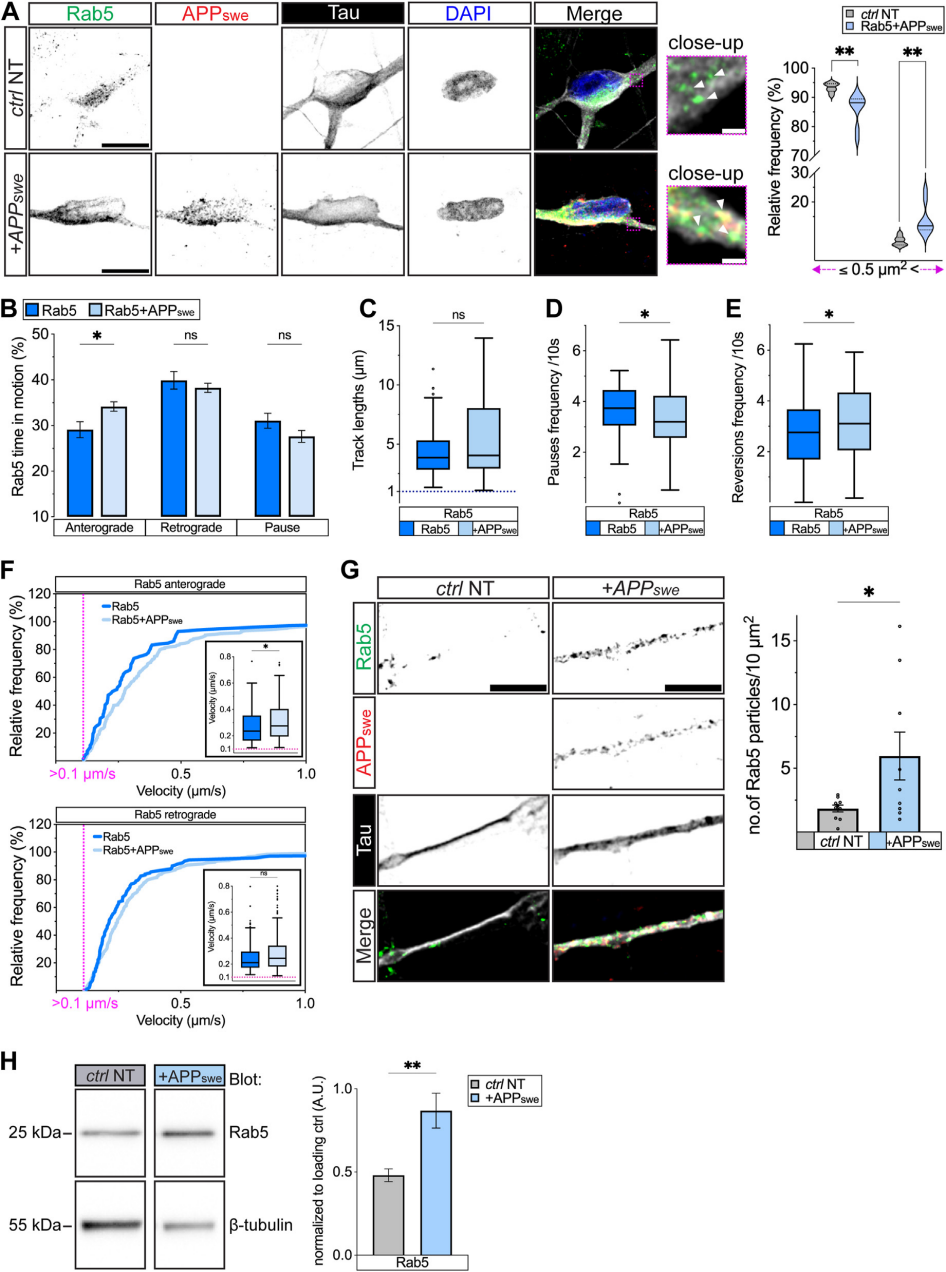
**Impaired axonal transport of Rab5+ early endosomes by the Swedish variant.***A*, representative micrographs showing nontransduced (NT) and APPswe transduced neurons stained against Rab5 (*green*), APP (*red*), tau (*white*), and DAPI (*blue*) (scale bar represents 10 μm). Close-ups with *white arrowheads* pointing at Rab5+ puncta of different sizes (scale bar represents 1 μm). Violin plots showing relative frequencies of Rab5+ puncta sizes binned into two subpopulations: smaller or equal (≥) and larger (>) than 0.5 μm^2^ (right, *n* ≥ 9 neurons analyzed per each condition from three biological replicates). *B*, percentage of Rab+ particles in anterograde and retrograde real-time motion or pausing in human neurons transduced with either Rab5 or Rab5+APPswe (*n* ≥ 70 particles from three biological replicates). *C*, track lengths (distances) of Rab5+ trajectories in Rab5 or +APPswe transduced human neurons (*n* > 45 particles from three biological replicates). *D*, 10 s pause frequency of Rab5+ particles in Rab5 or +APPswe transduced human neurons (*n* ≥ 70 particles from three biological replicates). *E*, 10 s reversions frequency of Rab5+ particles analyzed in axons over-expressing Rab5 or +APPswe (*n* ≥ 70 particles from three biological replicates). *F*, relative frequency of Rab5+ segmental velocities moving anterograde (*upper panel*) and retrograde (*lower panel*) quantified in Rab5 or Rab5+APPswe transduced human neurons. Velocities >0.1 μm/s are represented as cumulative frequency distributions. Subgraphs show segmental velocities distribution as box-and-whiskers (*n* > 70 segments from ≥ 70 particles analyzed from three biological replicates). *G*, representative immunofluorescences from non-transduced control (*ctrl* NT) and APPswe transduced neurons stained for Rab5 (*green*), APPswe (*red*), and tau (*white*) (scale bar represents 5 μm). Densities are calculated as the number of Rab5+ particles every 10 μm^2^ of distal neurites (at least 70 μm away from the soma, *n* ≥ 9 neurons per each condition from three biological replicates). *H*, representative immunoblots of Rab5 and βIII-tubulin bands (*left*), analyzed in *ctrl* NT or APPswe-transduced SH-SY5Y, carried out in the same experiment. Densitometric analysis (A.U. = arbitrary units) of Rab5 intensities from *ctrl* NT or APPswe samples normalized to βIII-tubulin loading control (*right*; *n* = 8 biological replicates). Full samples intensities and quantifications are shown in Fig. S10, *F* and *G*. Data are represented by 0 to 100 percentile violin plot (*A*), mean ± s.e.m. (*B*, *G*, and *H*), Tukey’s box-and-whisker plot (*C*, *D*, *E*, and subgraphs in *F*), cumulative frequency distributions (*F*) (∗*p* < 0.05, ∗∗*p* < 0.01). Statistical comparisons were performed using 2-way ANOVA followed by Šídák's multiple comparisons test (*A* and *B*), Mann-Whitney *U* test (*C*–*F*), and unpaired *t* test (*G* and *H*). APP, amyloid precursor protein.

To test this hypothesis, we initially focused on the movement of the early endosomes since they are at least in part transported by the same molecular motors as APP within the axons (48, 49), in addition to being implicated early and consistently in the pathophysiology of AD (50). Previous work showed increased size of Rab5-positive (Rab5+) endosomes in several cell culture paradigms mimicking aspects of AD pathology including human iPSCs harvested from patients carrying FAD variants (10, 51). To test whether our cell culture reproduces this endosomal phenotype (Fig. 6*A* - *left*), we transduced human neurons with APP_swe_ coupled to tRFP and stained the cultures for Rab5, tRFP, tau, and DAPI. In contrast to nontransduced cultures, neurons expressing APP_swe_ showed a significant increase in the size of the Rab5+ particles with a reduced number of endosomes with areas ≤0.5 μm^2^ and increased frequency of endosomes with areas > 0.5 μm^2^ (Fig. 6*A* – *right* graph).

To examine the effects of the Swedish variant on the axonal transport of Rab5+ endosomes, neurons were cotransduced with either APP_wt_ and RFP-Rab5 or with APP_swe_ and EGFP-Rab5 and the transport of Rab5+ particles measured (Movies S9 and S10). Comparison of hNSCs-derived neurons transduced with RFP-Rab5 or cotransduced with APP_wt_ and RFP-Rab5 showed no difference in any of the examined parameters of the Rab5+ transport using either net or segmental axonal transport analyses (Fig. S10, *A*–*E*). In contrast, neurons cotransduced with APP_swe_ and EGFP-Rab5 showed significantly increased time in anterograde motion of Rab5+ particles compared with Rab5-only transduced neurons with comparable track lengths (Fig. 6, *B* and *C*). These observations were accompanied by significantly increased reversions frequencies but no changes in pause frequencies of Rab5+ particles in neurons cotransduced with APP_swe_ and EGFP-Rab5 compared with Rab5-only transduced neurons (Fig. 6, *D* and *E*). Segmental velocities of anterogradely but not retrogradely moving Rab5+ particles were significantly increased by APP_swe_ cotransduction as well (Fig. 6*F*).

If the Swedish variant increases the amount of time and the speed by which Rab5 endosomes travel in the anterograde direction, then it should also increase their density in distal projections (Fig. 6*G* – *left*). Testing this hypothesis, we observed a significantly increased number of endogenous Rab5+ particles in APP_swe_ *versus* nontransduced distal projections (Fig. 6*G* – *right* graph). These results suggested that the Swedish variant causes the mislocalization of Rab5 endosomes into distal projections, which likely prevents their physiological maturation and leads to their aberrant accumulation. To test whether the Swedish variant leads to aberrant accumulation of Rab5 endosomes, we prepared lysates from nontransduced APP_wt_ or APP_swe_-transduced SH-SY5Y cells and carried out measurement of Rab5 levels all in the same experiment (Fig. 6*H* – *left*-ctrl NT and APP_swe_; Fig. S10*F*-all samples). The measurements noted significantly increased Rab5 levels in cultures transduced with APPs with APP_swe_ demonstrating the most significant increase compared with nontransduced cultures (Figs. 6*H* – *right*; S10*G*). These experiments reveal that the Swedish variant impairs endosomal axonal transport and identifies a subset of endosomes that pathologically reverse their axonal transport directionality and as a result, aberrantly accumulate in distal projections.

### Swedish pathogenic variant undermines retrograde axonal transport of lysosomes

**Figure 7:**
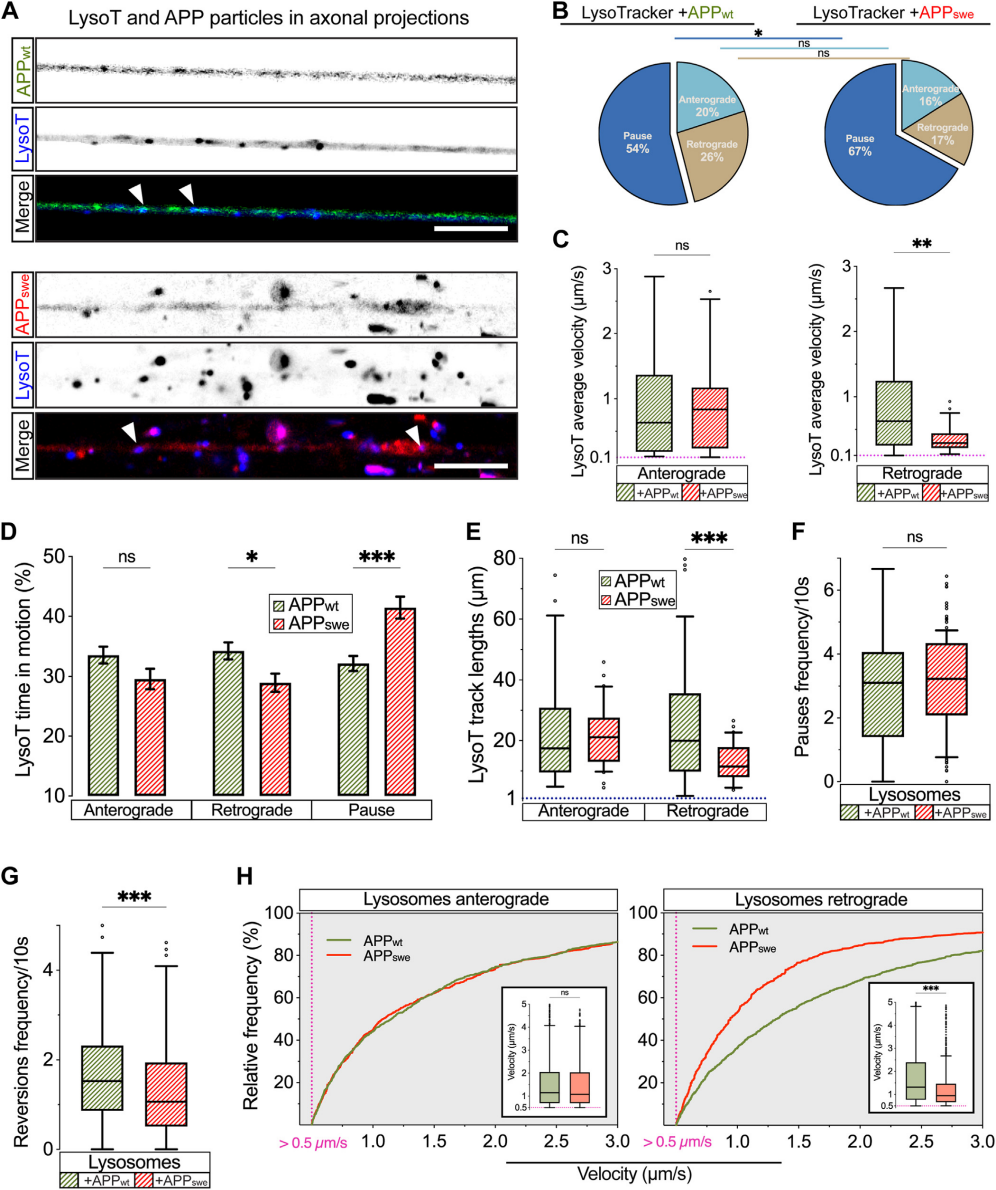
**Swedish FAD variant affects axonal transport of LysoT+ organelles.***A*, representative images of LysoTracker (LysoT) DeepRed (+) labeled lysosomes prior to 30 s time-lapse recordings in neurons transduced with either APP_wt_ (*up*) or APP_swe_ (*down*; scale bar represents 10 μm). *B*, proportions (%) of moving (anterograde or retrograde) and stationary (pause) LysoT+ organelles under either APP_wt_ or APP_swe_ transduction conditions (*n* = 17 axons from four biological replicates). *C*, average velocities of anterogradely or retrogradely moving LysoT+ organelles, in either APP_wt_ or APP_swe_ transduced neurons (*n* > 30 particles from four biological replicates). *D*, percent real-time motion, anterograde or retrograde, and pausing of LysoT+ organelles in APP_wt_- or APP_swe_-transduced neurons (*n* > 150 particles from four biological replicates). *E*, quantification of track lengths (distances) reached by either anterograde or retrograde LysoT+ organelles in both APP_wt_- and APP_swe_-transduced human neurons (n > 150 particles from four biological replicates). *F*, 10 s pause frequency of LysoT+ organelles quantified from both APP_wt_- and APP_swe_-transduced human neurons (*n* > 150 particles from four biological replicates). *G*, 10 s reversions frequency of LysoT+ organelles quantified from both APP_wt_- and APP_swe_-transduced human neurons (*n* > 150 particles from four biological replicates). *H*, relative frequency of LysoT+ organelles instantaneous velocities >0.5 μm/s from both APP_wt_- and APP_swe_-transduced human neurons. Subgraphs show instantaneous velocity distribution as box-and-whiskers (*n* > 900 velocity values from 17 axons and 4 biological replicates). Data are represented as proportions (cake plot-*B*), mean ± s.e.m. *D*, Tukey’s box-and-whisker plot (*C*, *E*, *F*, and *G* subgraphs *H*), and cumulative frequency distributions (*H*). Statistical comparisons were performed using 2-way ANOVA followed by Šídák's multiple comparisons test (*B* and *D*), Mann-Whitney *U* test (*C*, *E*, *F*, *G*, and subgraphs *H*) (∗*p* < 0.05, ∗∗*p* < 0.01, ∗∗∗*p* < 0.001). FAD, familial AD.

A wealth of data reports that a significant part of the early endosomes evolves into late endosomes and eventually fuses with lysosomes (52, 53, 54). As our previous experiments showed that the Swedish variant perturbs axonal transport of the early endosomes, we next tested whether it also impacts the transport of other compartments of the endosomal lysosomal pathway. To this end, the hNSCs-derived neurons were transduced with either APP_wt_ or APP_swe_ and concomitantly stained with the Lysotracker DeepRed (LysoT), to allow for the acquisition of the movies of the lysosomal axonal transport (Fig. 7*A*; Movies S11 and S12).

The net analysis of axonal transport showed a significantly increased proportion of stationary (Fig. 7*B*) and significantly reduced average velocities of the retrogradely but not of the anterogradely transported LysoT particles in APP_swe_ compared with APP_wt_-transduced neurons (Fig. 7*C*). In agreement with this net analysis, the segmental analysis demonstrated significantly decreased retrograde time in motion at the expense of significantly increased pausing time of the lysosomes in APP_swe_ compared with APP_wt_ axons (Fig. 7*D*). Track lengths of retrogradely but not of anterogradely transported LysoT particles were also decreased in APP_swe_ compared with APP_wt_ (Fig. 7*E*). In contrast to pause frequencies which showed no differences, reversion frequencies were significantly lower in APP_swe_ in comparison with APP_wt_ (Fig. 7, *F* and *G*). Last, when analyzing instantaneous velocities of LysoT particles faster than 0.5 μm/s, we found that retrograde but not anterograde velocities were also significantly slower in APP_swe_ compared with APP_wt_ axons (Fig. 7*H*).

These experiments show that the Swedish variant significantly perturbs the physiologically predominant retrograde axonal transport of lysosomes. Altogether, they also suggest that the Swedish variant hinders the biology of the entire endosomal lysosomal pathway.

## Discussion

**Figure 8:**
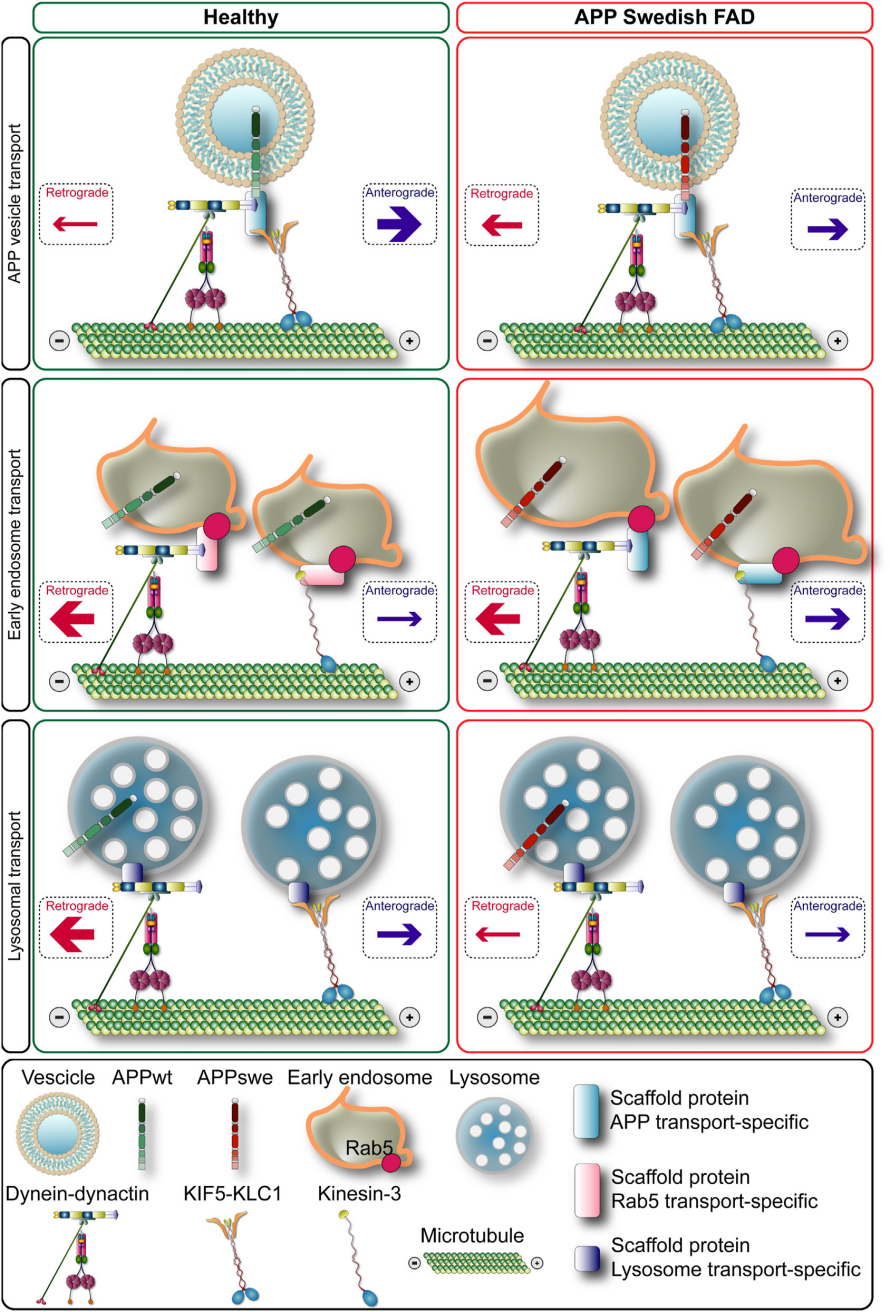
**Proposed model by which Swedish FAD variant perturbs axonal transport of the APP vesicles and the endosomal-lysosomal system.** Model: *top* – Swedish variant reduces the canonically higher proportion of anterogradely transported APP vesicles and enhances the association between DCTN1 and APP_swe_, consequently promoting inefficient bidirectional movement. *Middle* – Swedish variant affects the transport of Rab5+ early endosomes, which become enlarged and aberrantly accumulate in distal regions of the axons. *Bottom* – Swedish variant perturbs physiological retrograde axonal transport of lysosomes by reducing their retrograde velocities and increasing their stationary proportion. The presented components of the cargo molecular motor assemblies are merely illustrative for the purpose of showing the effects of the pathogenic Swedish variant on axonal transport. APP, amyloid precursor protein; FAD, familial AD.

Mounting evidence suggests that impaired axonal transport contributes causally or otherwise to the pathogenesis of AD (13, 55). Early experiments showed that animal models carrying FAD pathogenic variants exhibit decreased APP levels at the proximal stump of ligated sciatic nerves and reduced Mn^2+^ and NGF transport in the hippocamposeptal pathway (35, 36, 56, 57). These experiments indicated a role of APP in perturbing axonal transport but failed to discriminate between the effects of the overexpression of WT *versus* pathogenic APP variants. This was later clarified with cell culture experiments, which showed that pathogenic variants as well as the overexpression of β−CTFs or blockage of β- and γ-cleavage sites of APP all impair axonal transport (37, 46). It is well-established that pathogenic APP variants perturb endosomal structure and function, which is affected early and consistently in AD (10, 51). Aberrant accumulation of vesicular structures consistent with morphologically altered endosomes and lysosomes has also been observed within axonal swellings in animal models of AD (34). The observation of these vesicular structures together with recent studies provide preliminary evidence of impaired axonal transport of endosomes and lysosomes by pathogenic FAD variants (47, 58, 59). Building on these studies, we here show that impairment in axonal transport by the Swedish APP variant involves not only anterograde but also retrograde axonal transport machinery and causes more widespread derangement of the axonal transport pathways than previously thought (Fig. 8 - *top*).

We observed that the Swedish APP variant causes a significant standstill of the APP particles physiologically destined to be transported distally within axons at the expense of their overall reduced anterograde axonal transport. This observation indicates that the Swedish APP variant acquires features typical of bidirectional movement with altered coordination of the actions of the anterograde and retrograde molecular motors, which is reminiscent of dysfunctional processivity of the cargo motor assemblies (60, 61). In agreement with this observation is the finding that the Swedish variant perturbs the association between APP and DCTN1. Considering that DCTN1 is fundamental for processive motility of the retrograde motor dynein (21), recruiting more DCTN1 to the cellular compartments harboring APP is predicted to enhance the activation of the retrograde machinery and thus counteract the anterograde movement. These findings are consistent with previously reported abnormalities in the dynein–DCTN1 complex in aging and AD (22, 27, 33).

Here presented experiments show that blockage of the APP β-cleavage site does not affect the association between APP and DCTN1 (37). This suggests that the association between APP and DCTN1 occurs independently from the effect of the Swedish variant on the β-cleavage of APP. Considering that we have shown that FAD variants perturbing β- and γ-cleavage sites of APP both impair axonal transport, the lack of changes in the association between APP and DCTN1 following BACE inhibition is not entirely surprising. Mindful of the complexity of the proteolytic processing of APP and the regulatory processes of the motor machinery including a battery of critical adaptors such as the GSK3β and the JUN N-terminal kinase–interacting proteins among others (62, 63, 64, 65), further work is required to critically decipher the mechanisms by which pathogenic FAD variants alter the association between APP and its molecular motors to impair axonal transport.

The changes identified in the transport of early endosomes and lysosomes indicate that the Swedish variant gives rise not only to impaired axonal transport of APP but to a significantly more widespread derangement of axonal pathways (59). Intriguingly, the Swedish variant reverses the physiological predominantly retrograde axonal transport of a subset of early endosomes by favoring their anterograde movement (Fig. 8 - *middle*). This explains the aberrant accumulation of early endosomes distally in the axons where being misplaced, the endosomes cannot mature into lysosomes. Not surprisingly, lysosomes are also impacted by the Swedish variant since not all undergo their physiological retrograde axonal movement (Fig. 8 - *bottom*). These findings raise a timely question of whether impairments in axonal transport of the endosomal-lysosomal system underlie or play an otherwise contributory role in their enlargement and malfunction in AD (66).

Our findings fuel the hypothesis that impairments in axonal transport promote axonal pathology and pathogenesis of AD (12, 13, 67, 68). Genetic manipulations of APP (34), its proteolytic machinery (32, 35, 69), and of all of the other major proteins linked to AD including tau (70, 71) and ApoE (72) in flies and mice invariably produce axonal pathology reminiscent of the one observed in AD and described in molecular motor deficiencies (73). Our findings, together with these studies, suggest that perturbed coordination of the actions of the molecular motors underlies axonal transport defects and ultimately translates into axonal pathology in AD. While previous and our study of the rare pathogenic FAD variants suggest a role of APP and thus of axonal cargos in causing axonal impairments, the mechanisms underlying axonal pathology in the most common sporadic form of AD remain largely unknown. Recently described abnormalities in kinesin and dynein-DCTN1 in cell and animal models as well as in the brain of patients with sporadic AD (22, 24, 25, 27, 31, 33, 74) suggest that changes in molecular motors rather than in axonal cargoes may be responsible for the axonal transport impairments in sporadic AD. The observation that molecular motors undergo significant changes during aging (24, 25, 33, 74), which is the major risk factor for sporadic AD (26), provides further support for the role of molecular motor abnormalities in axonal pathology in sporadic AD. In conclusion, we are hopeful that our study of the rare Swedish APP variant represents a constructive contribution to elucidating the mechanisms underlying axonal transport defects in AD and also to our understanding of the axonal impairments in other neurodegenerative disorders manifesting with axonal pathology (75).

## Experimental procedures

Detailed methods and materials are provided in *SI Experimental Procedures*. In this section are listed all the cell lines used in the study, including details about differentiation into neurons (in the case of H9-derived NSCs), neuronal-like cells (in the case of SH-SY5Y), or neural progenitors (in the case of iPSCs derived from a healthy donor and a FAD APP_swe_ patient), and animal models.

Briefly, H9-derived NSCs were differentiated for 40 DIV and first used for axonal transport experiments to detect APP movement (APP_wt_, APP_swe_, and APP_lon_ conjugated with different tags) and Rab5 and lysosome movement. Axonal transport analysis was performed by recording movies of particles at 2fps or 4fps for a total of 30 s and 60 s, respectively, using a confocal microscope equipped with a live module (Zeiss Confocal LSM780, Zeiss Live LSM7) and an immersion oil objective 63x/1.4 NA Plan Apochromat. The parameters of axonal transport were analyzed by using the spot tracking algorithm package of Imaris software (https://imaris.oxinst.com, Oxford instruments).

Second, we used human neuronal cultures for all the immunocytochemistry experiments, which we performed to establish pNFH and MAP2 expression levels, for axonal projections identification (pNFH and GFP staining), for APP_wt_ *versus* APP_swe_ distribution, and to investigate the Rab5 puncta size and densities. Images were acquired by using either an inverted Zeiss LSM 780 (Zeiss) or a Leica DM 6000B (Leica Microsystems) confocal microscope.

On the other hand, for part of the biochemical studies where we investigated the interaction of APP with different motor proteins and adaptors, we used SH-SY5Y cells, which were differentiated using a re-adaptation of the protocol published by Shipley *et al.* (2016). Twenty DIV SH-SY5Y cells were transduced with either APP_wt_ or APP_swe_ prior to performing IPs and analyses with SDS-PAGE/Western blot. Additionally, for our biochemical analyses, we used neural progenitors from iPSCs of a healthy subject (cv-hiPSC karyotype XY SD2010-125, UCSD) and a FAD APP_swe_ patient (APP_swe_ Kp9-hiPSC) (39). All the details about amplification and differentiation protocols are available in the SI Experimental Procedures, which were performed as published by Holubiec *et al.* (2023). For both SH-SY5Y and human neural progenitors, protein extraction, IPs, SDS-PAGE, and Western blotting were performed with the same method and the same materials: magnetic beads (for IP), buffers, antibodies (Table S1) are all listed in detail in the SI Experimental Procedures.

For the evaluation of APP and dynactin-1 levels in mouse brains, we used both WT and APP_swe_/PS1 M146V animal models obtained from PsychoGenics, handled in compliance with the Association for Assessment and Accreditation of Laboratory Animal Care and approved by the Institutional Animal Care and Use Committee under the National Institute of Health Guide for the Care and Use of Laboratory Animals. Sagittal sections of the brains were cut and stained for dynactin-1, APP, MAP2, and DAPI. Entire brain sections were imaged on a Zeiss AxioScan.Z1 slide scanner microscope at 10X magnification and then analyzed by using Image Pro Premier (v9.1 or higher). Additional details about IHC protocol and analysis can be found in the SI Experimental Procedures.

All the statistical analyses were performed using GraphPad Prism 10.0; details about comparisons can be found in the figure legends.

## Data availability

All relevant data associated with the current study are available in the manuscript or the supplementary material.

Additional information about data and protocols is available from the authors upon request.

## Supporting information

This article contains supporting information (38).

## Conflict of interest

The authors declare no that they have no conflicts of interests with the contents of this article.
