# Supplementary figures and images for: Swedish Alzheimer’s disease variant perturbs activity of retrograde molecular motors and causes widespread derangement of axonal transport pathways
Source: J Biol Chem. 2024 Mar 5;300(4):107137. doi: 10.1016/j.jbc.2024.107137 (PMC10997842; doi:10.1016/j.jbc.2024.107137)

# Supplementary Figure 1 - Feole et al.

**A**

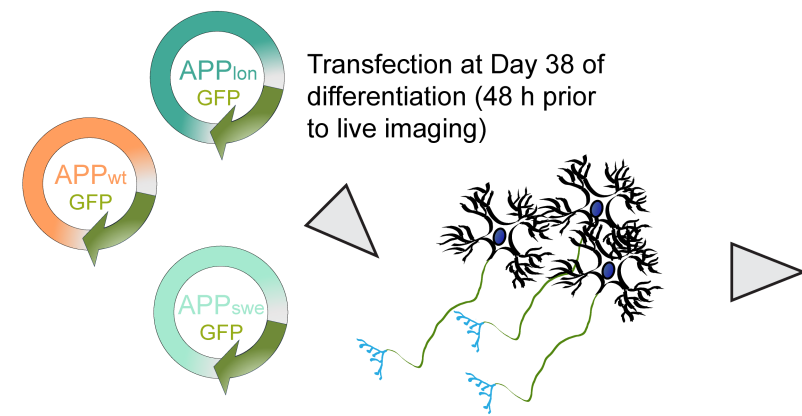

Live-imaged neurites

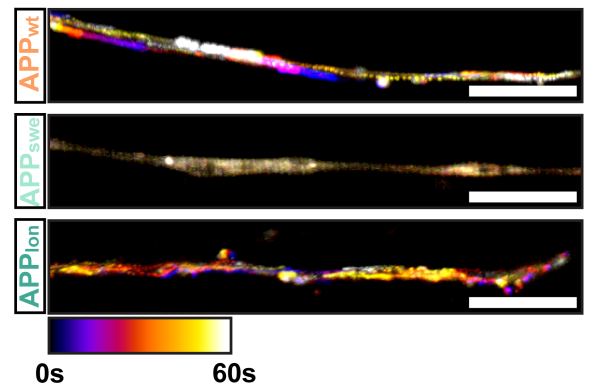

**B**

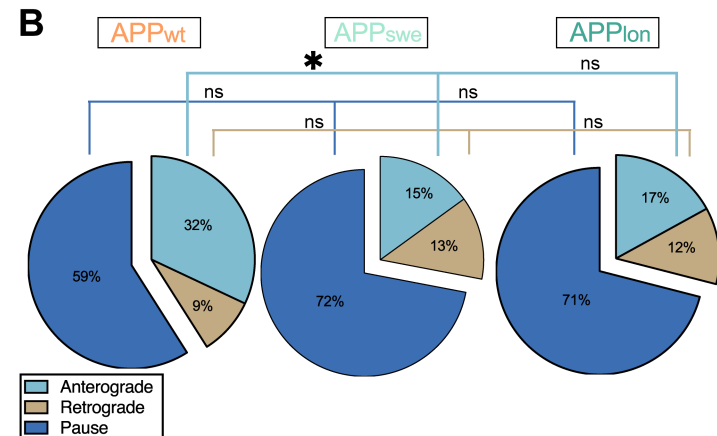

**C**

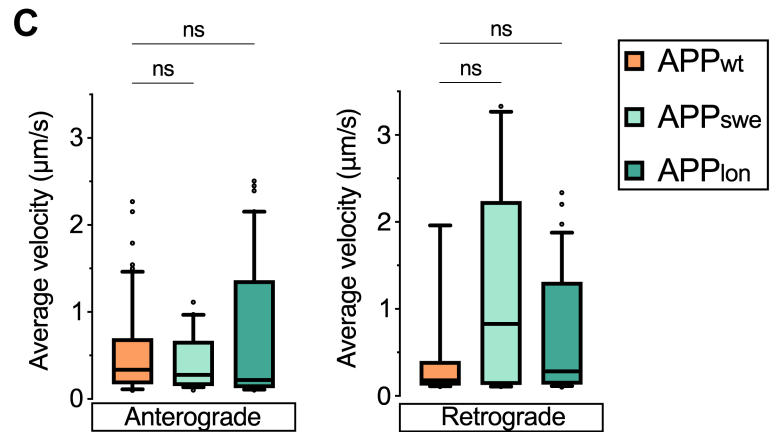

**D**

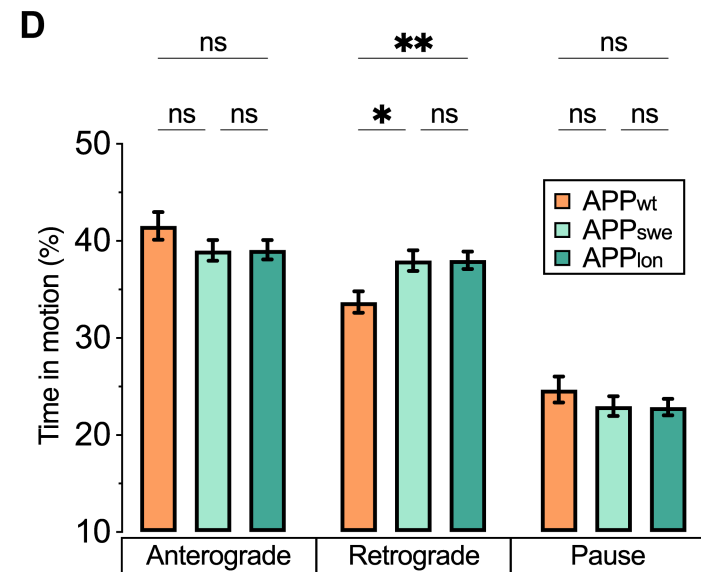

**E**

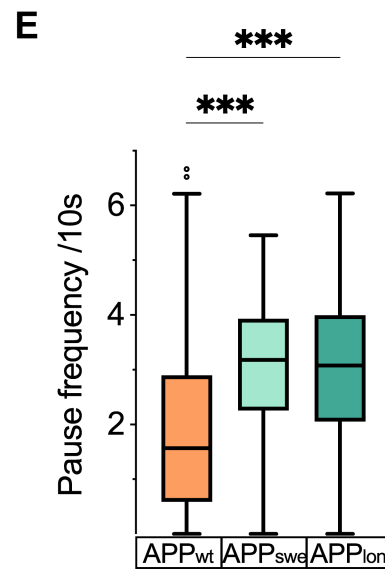

**F**

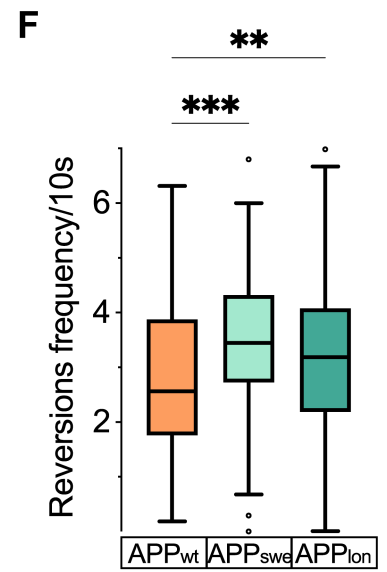

Supplement: Supporting Figure S1 [file mmc13.pdf]

A

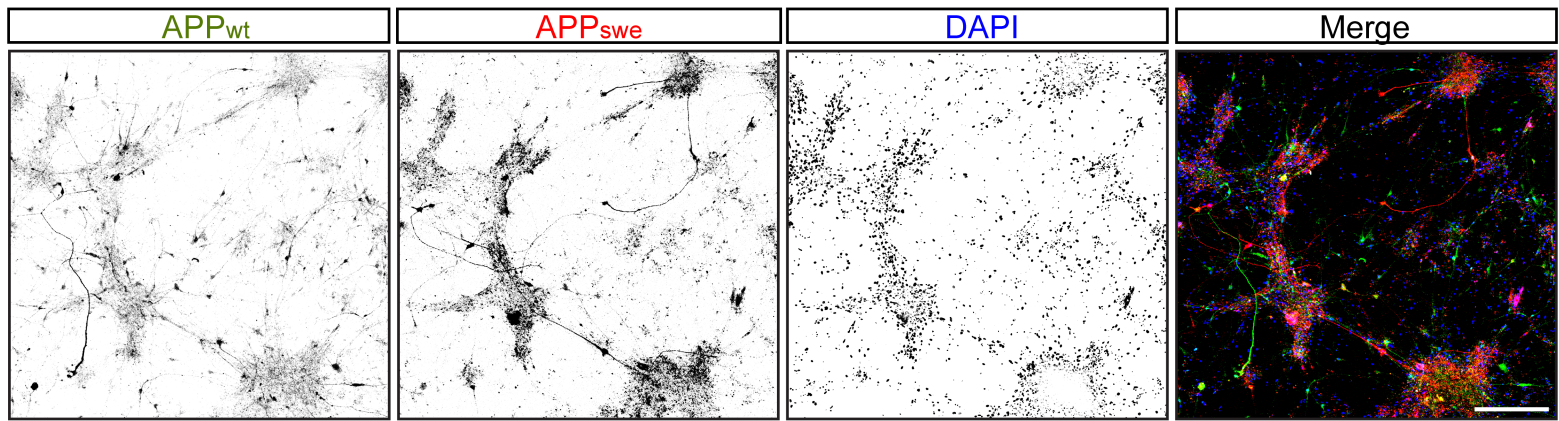

B

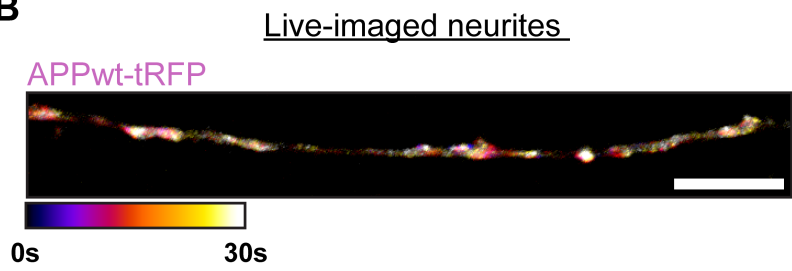

C

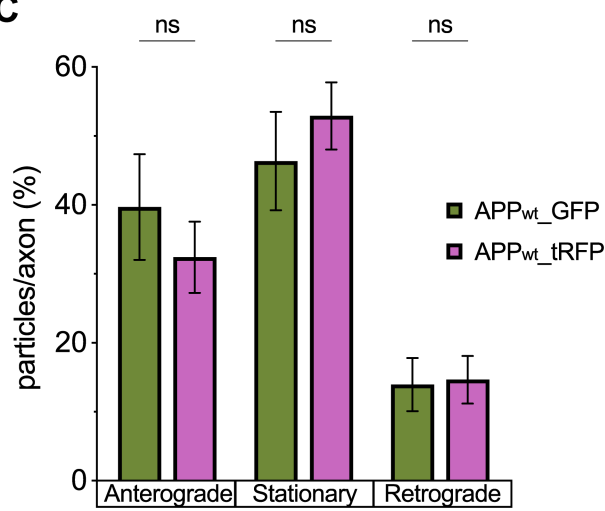

Supplement: Supporting Figure S2 [file mmc14.pdf]

Supplementary figure 3 - Feole et al.

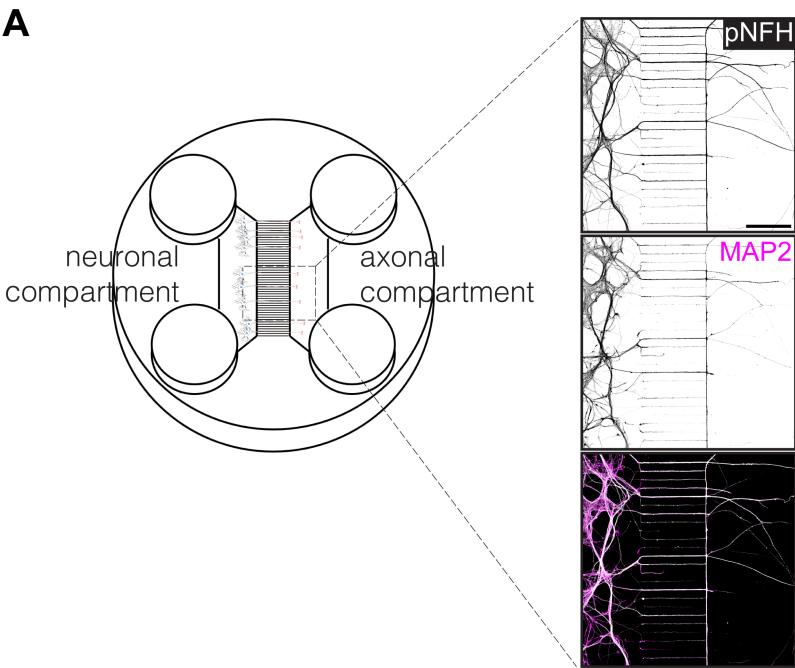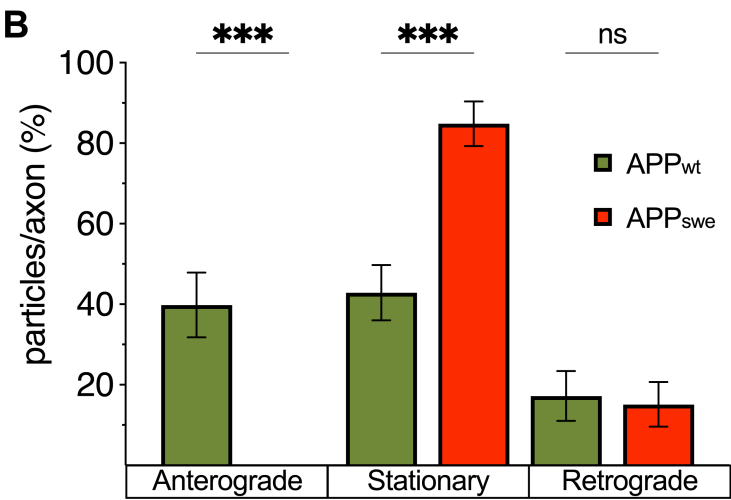

Supplement: Supporting Figure S3 [file mmc15.pdf]

# Supplementary figure 4 - Feole et al.

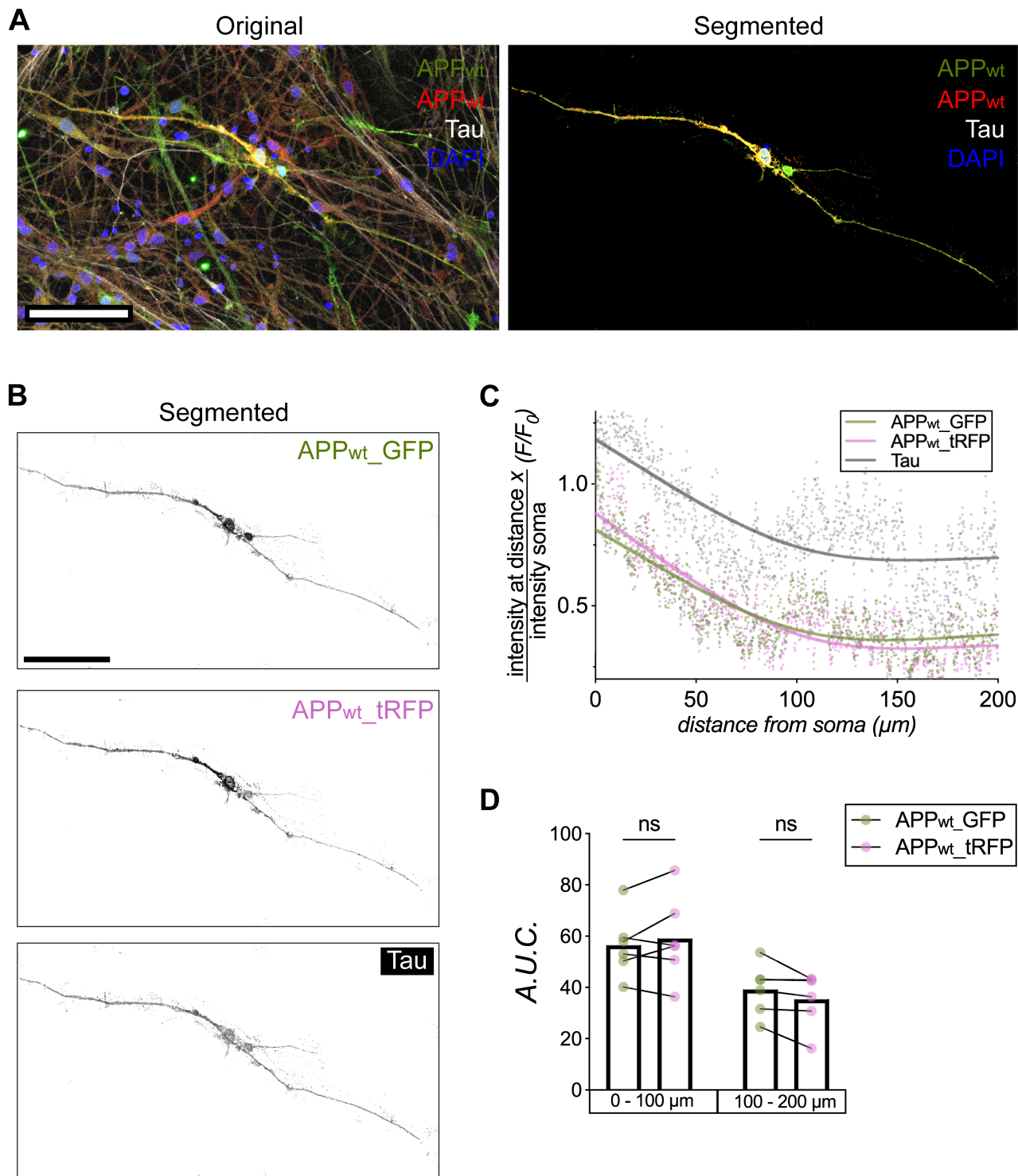

Supplement: Supporting Figure S4 [file mmc16.pdf]

Supplementary Figure 5 - Feole et al.

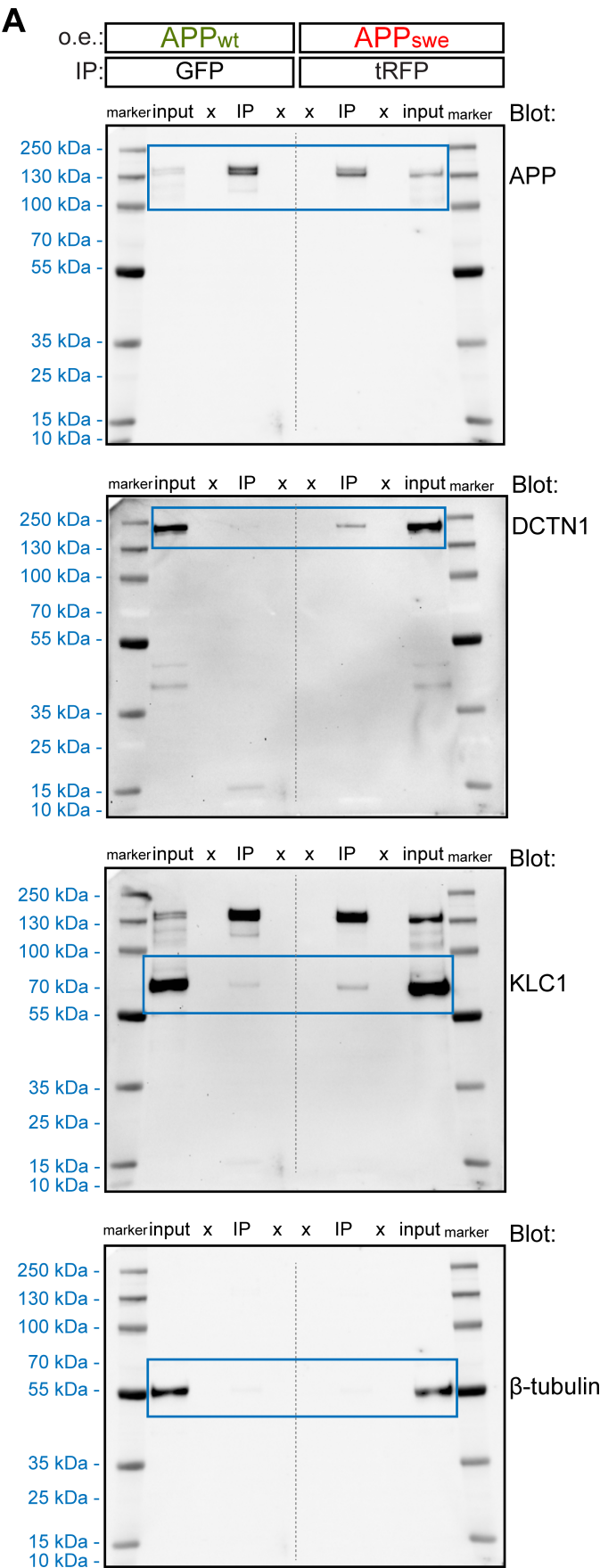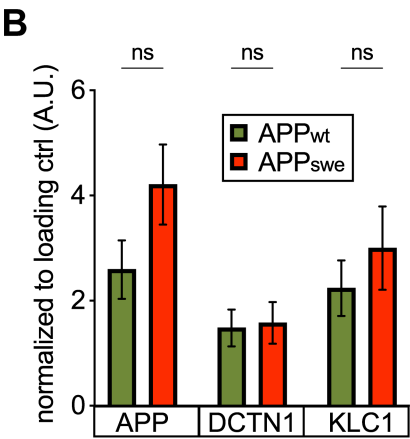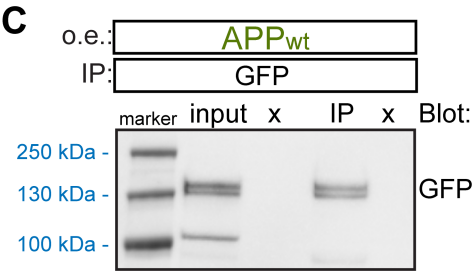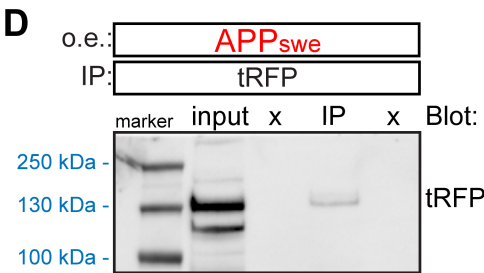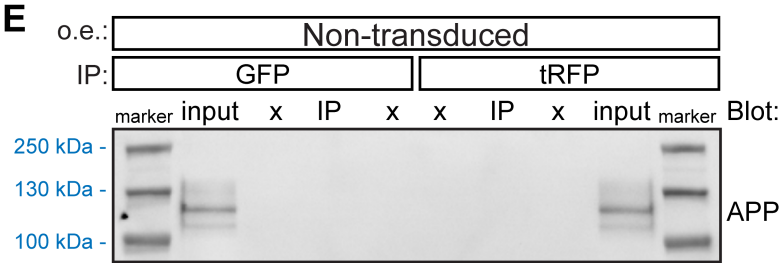

Supplement: Supporting Figure S5 [file mmc17.pdf]

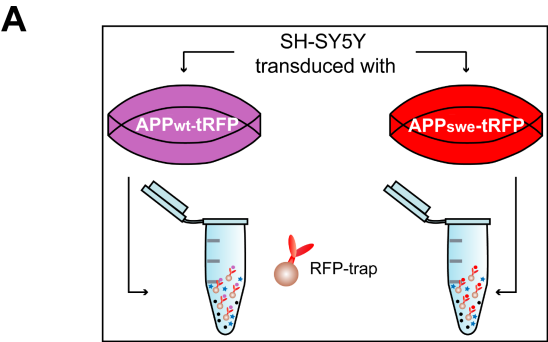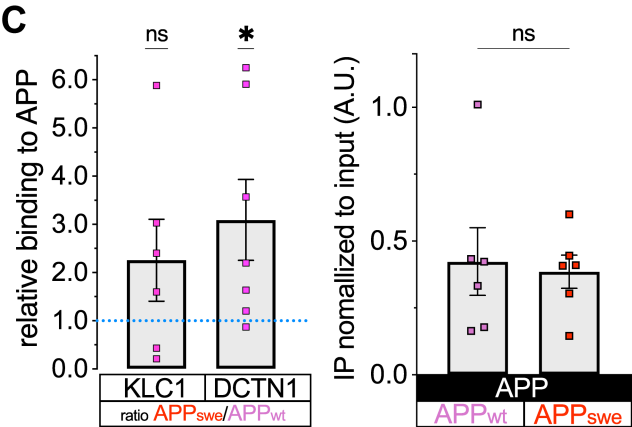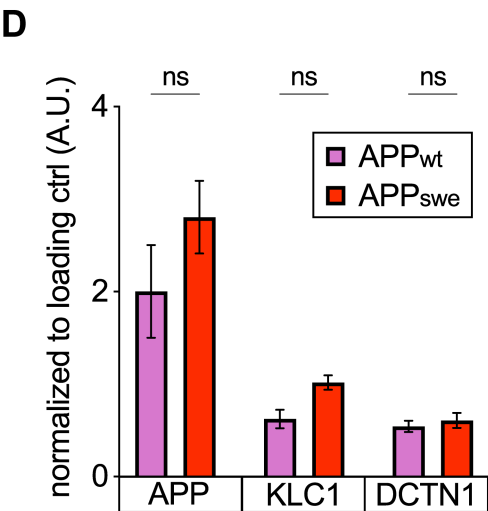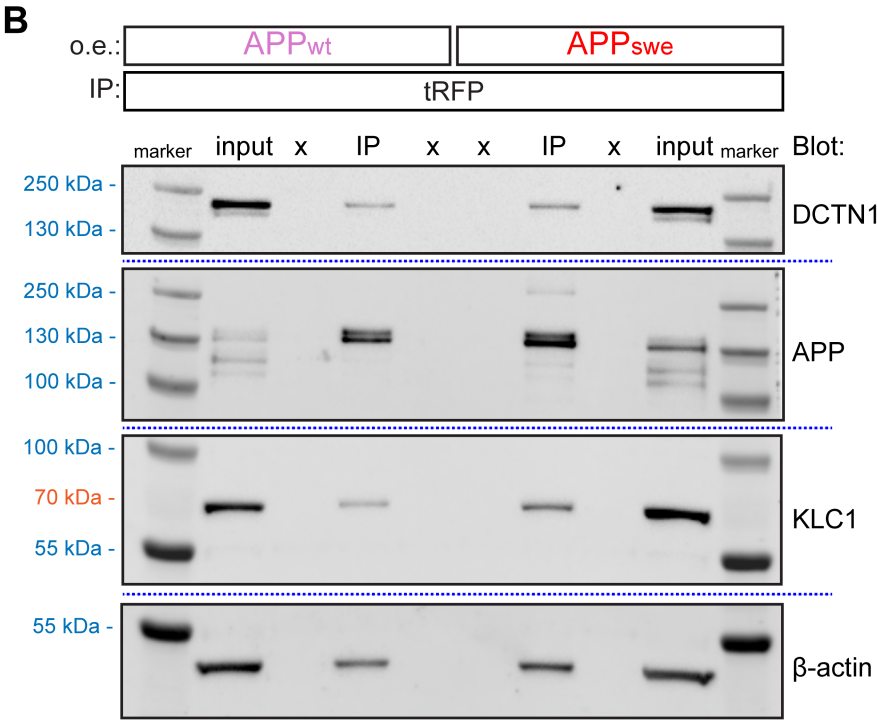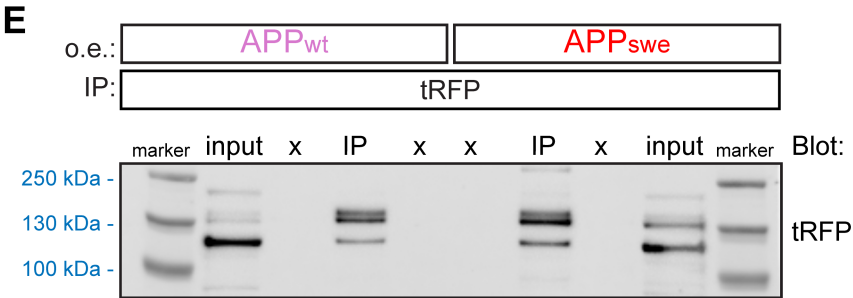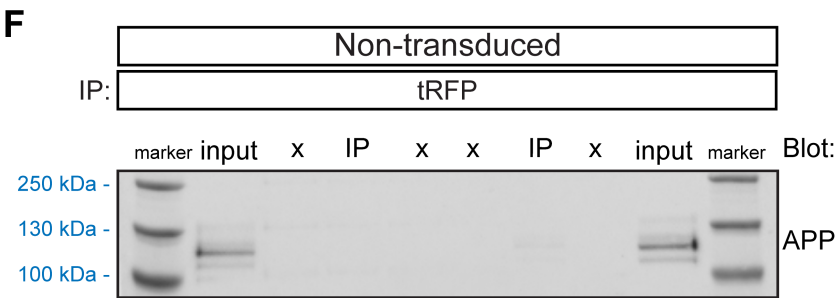

Supplement: Supporting Figure S6 [file mmc18.pdf]

Supplementary Figure 7 - Feole et al.

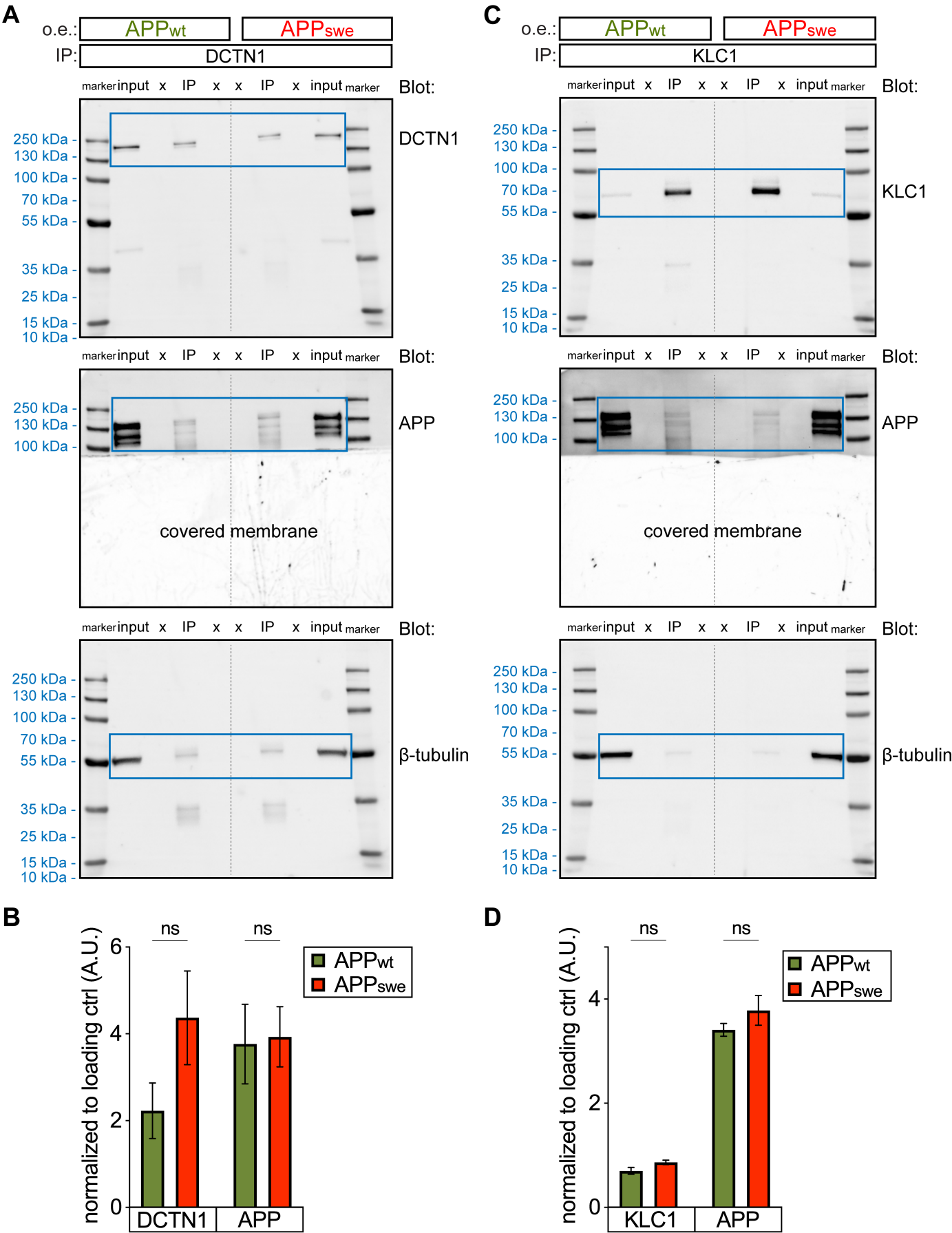

Supplement: Supporting Figure S7 [file mmc19.pdf]

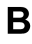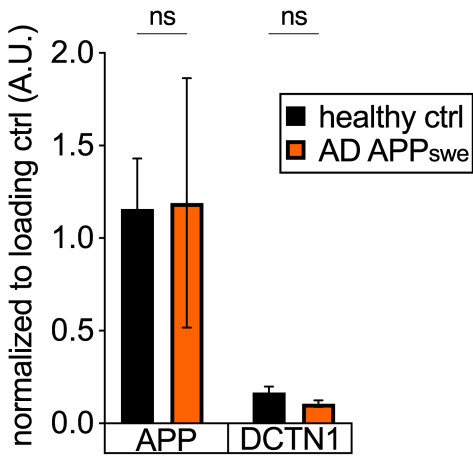

Supplement: Supporting Figure S8 [file mmc20.pdf]

Supplementary Figure 9 - Feole et al.

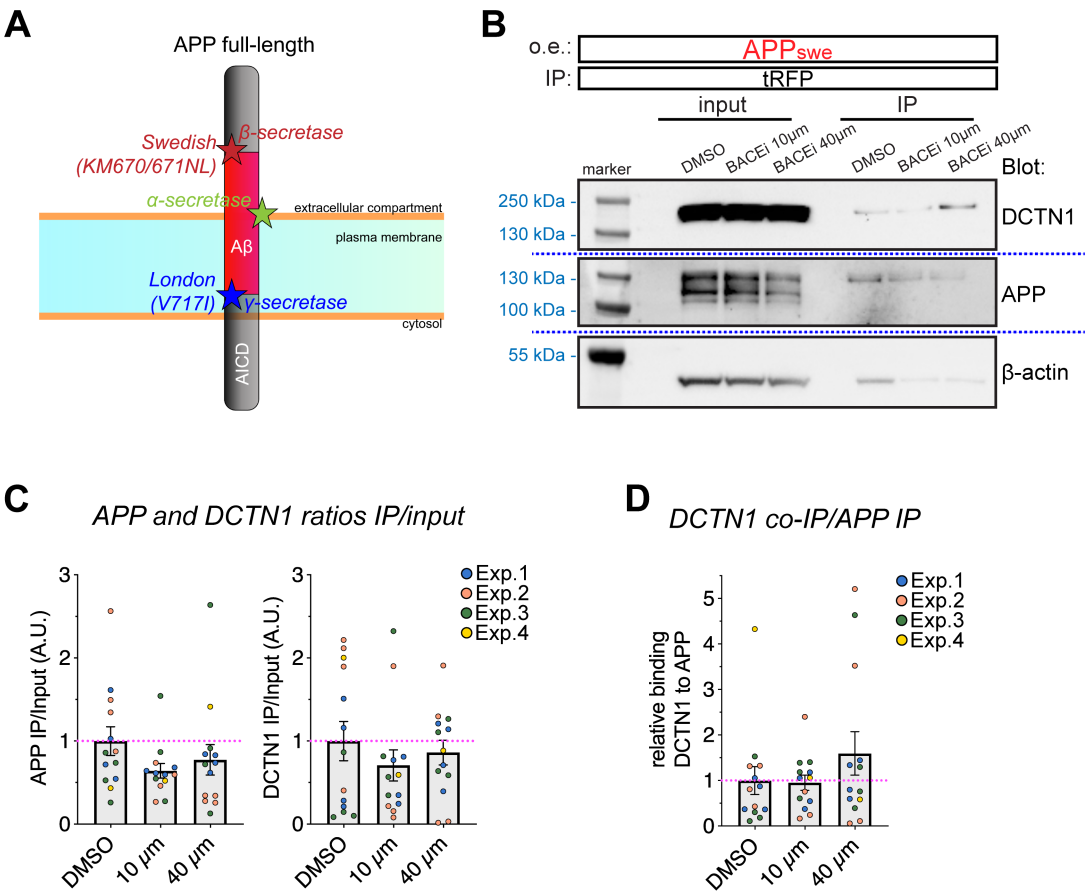

Supplement: Supporting Figure S9 [file mmc21.pdf]

Supplementary Figure 11 - Feole et al.

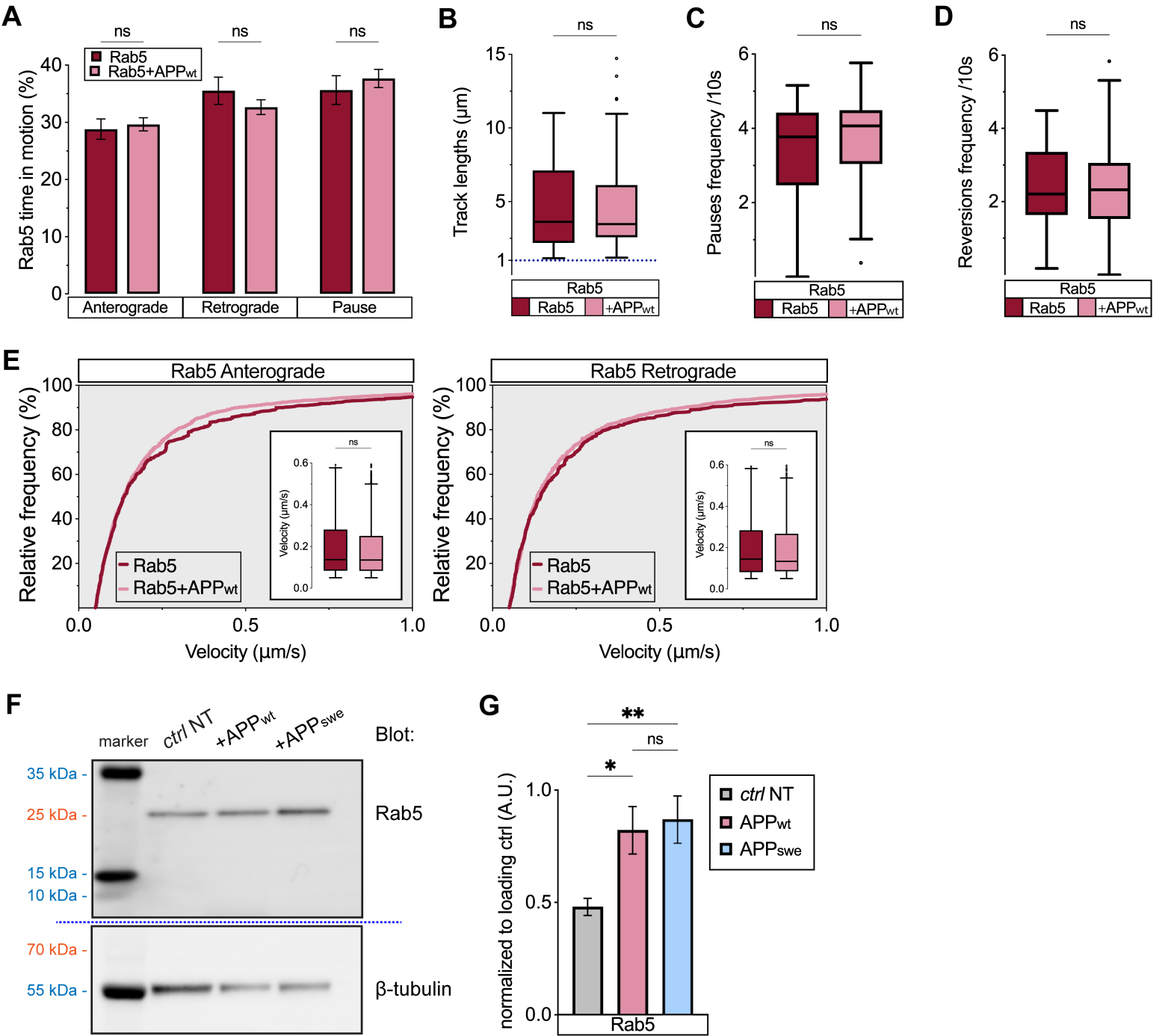

Supplement: Supporting Figure S10 [file mmc22.pdf]
