# Supplementary material for: Swedish Alzheimer’s disease variant perturbs activity of retrograde molecular motors and causes widespread derangement of axonal transport pathways
Source: J Biol Chem. 2024 Mar 5;300(4):107137. doi: 10.1016/j.jbc.2024.107137 (PMC10997842; doi:10.1016/j.jbc.2024.107137)
Supplement: Supporting Table S1 [file mmc23.pdf]

Antibodies	concentration	Source (cat. No.)
pNFH (phosphorylated Neurofilament Heavy Chain)	1:2000	BioLegend, 801601
MAP2 (Microtubule Associated Protein 2)	1:3000	Abcam, ab221693
MAP2 (Microtubule Associated Protein 2)	1:500	Sysy, 188004
MAPT (Microtubule Associated Protein tau)	1:400	Abcam, ab62639
tRFP (turbo red Fluorescent Protein)	1:1000	Evrogen, ab233
GFP (Green Fluorescent Protien)	1:1000	Abcam, ab5440
GFP (Green Fluorescent Protien)	1:1000	Abcam, ab1218
Rab5 (Ras-related protein Rab5)	1;1000	Cell signaling, #46449
p150 (Dynactin subunit 1)	1:50/1:1000	Cell signaling, #69399
p150 (Dynactin subunit 1)	1:200/1:100	Santa cruz biotechnology, sc-135890
APP (Amyloid Precursor Protein)	1:1000	Abcam, ab126732
APP (Amyloid Precursor Protein)	1:50/1:500/1:250	Abcam, ab32136
APP (Amyloid Precursor Protein)	1:1000	BioLegend, 803001
KLC1 (Kinesin Light Chain 1)	1:50/1:1000	Abcam, 174273
KLC1 (Kinesin Light Chain 1)	1:200	Santa cruz biotechnology, sc-58776
β-III tubulin	1:6000	BioLegend, 802001
β-actin	1:3000	Sigma-Millipore, A2228
Rabbit IgG isotype control	1 µg/µl	Novus biologicals, NB810-56910
Secondary antibodies used in ICC and IHC		
Alexa Fluor 488 donkey anti-mouse/rabbit/goat	1:500	Thermo Fisher, A31570,A21206,A11055
Alexa Fluor 546 donkey anti-mouse/rabbit/goat	1:500	Thermo Fisher, A10036,A10040,A11056
Alexa Fluor 555 donkey anti-mouse/rabbit/goat	1:500	Thermo Fisher, A31570,A31572,A21432
Alexa Fluor 647 donkey anti-mouse/sheep	1:500	Thermo Fisher, A31571, A21448
DyLight 647 donkey anti-mouse	1:500	Abcam, ab150107
Alexa 488 goat anti-guinea pig	1:500	Abcam, ab150185
Secondary antibodies used in western blot		
HRP-conjugated anti-Rabbit	1:2000	Cell signaling, #7074
HRP-conjugated anti-Mouse	1:2000	Cell signaling, #7076
HRP-conjugated anti-Goat	1:2000	Santa cruz biotechnology, sc-2354
Clean-Blot IP detection reagent	1:200	Thermo Fisher, 21230
