# Supplementary material for: Swedish Alzheimer’s disease variant perturbs activity of retrograde molecular motors and causes widespread derangement of axonal transport pathways
Source: J Biol Chem. 2024 Mar 5;300(4):107137. doi: 10.1016/j.jbc.2024.107137 (PMC10997842; doi:10.1016/j.jbc.2024.107137)
Supplement: Supporting Information [file mmc24.docx]

**SUPPORTING INFORMATION**

**Swedish Alzheimer’s disease variant perturbs activity of retrograde molecular motors and causes widespread derangement of axonal transport pathways**

Monica Feole^1,2,3^, Victorio M. Pozo Devoto^1^, Neda Dragišić^1^, Cayetana Arnaiz^4^,

Julieta Bianchelli^4^, Kateřina Texlová^1,5^, Kristina Kovačovicova^5^, Jan S. Novotny^1,6^,

Daniel Havas^5^, Tomas L. Falzone^4,7^, Gorazd B. Stokin^1,6,8,9 *°^

^1^ Translational Ageing and Neuroscience Program, Centre for Translational Medicine, International Clinical Research Centre, St. Anne’s University Hospital, Brno, Czech Republic

^2^ Faculty of Medicine, Department of Biology, Masaryk University, Brno, Czech Republic.

^3^ School of Cardiovascular and Metabolic Medicine & Sciences, King's College London, London SE5 9NU, UK

^4^ Instituto de Investigación en Biomedicina de Buenos Aires (IBioBA-CONICET-MPSP), Partner Institute of the Max Planck Society, Buenos Aires, Argentina

^5^ PsychoGenics, 215 College Road Paramus, NJ 07652, USA

^6^ Institute for Molecular and Translational Medicine, Faculty of Medicine and Dentistry, Palacký University Olomouc, Olomouc, Czech Republic.

^7^ Instituto de Biología Celular y Neurociencia IBCN (UBA-CONICET), Facultad de Medicina, Universidad de Buenos Aires, Buenos Aires, Argentina

^8^ Division of Neurology, University Medical Centre, Ljubljana, Slovenia

^9^ Department of Neurosciences, Mayo Clinic, Rochester, MN, USA.

*Corresponding author e-mail: [gbstokin@alumni.ucsd.edu](mailto:gbstokin@alumni.ucsd.edu) (G.B.S.)

° Current address: Institute of Molecular and Translational Medicine, Faculty of Medicine and Dentistry, Palacky University, Olomouc, Czech Republic and Department of Neurology, Royal Gloucester Hospital, Gloucestershire NHS Foundation Trust, Gloucester, UK

SI EXPERIMENTAL PROCEDURES

Differentiation of human neuronal stem cells

Human Neural Stem Cells (hNSCs) derived from the NIH approved H9 (WA09) human embryonic stem cell line were purchased from Merck (Germany). The hNSCs were plated on matrigel-coated 100 mm Petri dishes and maintained in culture with NSCs expansion media (KO DMEM/F12, 2% StemPro Neural Supplement, 1% Glutamax, 20 ng/ml β-FGF, 20 ng/ml EGF), which was exchanged every second day (DIV 0-3). Upon reaching confluency, the cells were grown in the neural progenitors differentiation media (DMEM/F12, 1% B27, 0.5% N2, 1% Glutamax), which was exchanged every other day until DIV 9. Neural progenitors were then detached with accutase and centrifuged at 300xg for 5’ at RT and pellets resuspended in Neuronal Optimized Media complete (NOMc) (DMEM-F12, 2% B27, 1% N2, 1 µg/ml laminin, 100 nM cAMP, 200 ng/ml ascorbic acid, 10 ng/ml BDNF, 10 ng/ml GDNF, 10 ng/ml IGF). Cells were counted and then seeded at different densities depending on the experimental needs. Neurons were differentiated by changing the NOMc every 6 days up to DIV 40.

**Differentiation of human induced pluripotent stem cells**

Human induced pluripotent stem cells (HiPSCs) generated from a healthy subject (cv-hiPSC karyotype XY SD2010-125, UCSD) and a subject affected by AD due to the Swedish mutation (APPswe Kp9-hiPSC)[^40^](#_ENREF_40) were cultured in 100 mm Petri dish (p100) adherent plates (Corning) using a feeder layer of irradiated E13 mouse embryonic fibroblast (iMEF) plated onto gelatin-coated (0.1% Gelatin) plates 24 h before seeding iPSCs. Cells were grown in Dulbecco’s Modified Eagle’s KO Medium (KO-DMEM) supplemented with 10% Knockout Serum Replacement (KSR), 2 mM non-essential amino acids, 2 mM L-glutamine, 100 U/ml penicillin/streptomycin and 4 ng/ml of β-FGF (Human Embryonic Stem Cell Medium (HES), at 37°C and 5% CO_2_. Neural rosette formation was achieved using a previously described protocol (Fernandez Bessone, JON 2022). Small and even-sized colonies were enzymatically detached from their feeder layer using Collagenase IV (1mg/ml) (Gibco) for 15-40 min at 37°C. Cell aggregates obtained from one p100 culture plate were suspended in 10 ml of expansion medium (without β-FGF) in a T75 culture flask for 2 days at 37°C and 5% CO_2_. During this period cell aggregates acquired a spherical shape. The third-day medium was replaced with 10 ml Neural Induction Medium (NIM), comprised of an advanced DMEM/F12 supplemented with non-essential amino acids (1:100, Invitrogen), N2 supplement (1:100, Gibco), and 1 mg/ml Heparin. Cells were incubated at 37°C and 5% CO_2_. After 5 days, neural inducted cell aggregates were collected and used for total protein lysates preparation and immunoprecipitation experiments.

**Differentiation of human SHSY-5Y cells into a neuron-like population**

SH-SY5Y (ATCC, USA) cells were differentiated from a neuroblastoma-like state into a human neuronal-like cell culture. For our experiments, we readapted a well-established differentiation protocol (Shipley et al 2016). In brief, SH-SY5Y were seeded into 60 mm Petri dishes previously coated for 15 min with 0.1% Gelatin/PBS. Cells were maintained in DMEM complete (DMEM-high glucose, 10% Fetal Bovine Serum, 1% L-glutamine, 1% P/S) up to 80% confluency (DIV 0-2). Afterward, cells were passed into new 60 mm dishes, and the culture medium was switched to serum-free Optimem containing 1% B27, 10 ng/ml BDNF, 10 ng/ml cAMP, 10 µM RA, 1% P/S, 1% Glutamax and changed every second day up to DIV 20 when the cells were used for the experiments.

**Animal models**

APP/PS1 (C57BL/6NTac.CBA-Tg (Thy1-PSEN1*M146V, -APP*Swe)10Arte)(Taconic) and wild-type (WT, C57BL/6N) mouse brain sections were obtained from PsychoGenics (Tarrytown, NYC, US). Animals were handled in compliance with the Association for Assessment and Accreditation of Laboratory Animal Care (AAALAC). All procedures were approved by the Institutional Animal Care and Use Committee (IACUC) under the National Institute of Health Guide for the Care and Use of Laboratory Animals.

**Microfluidic chambers**

The microfluidic chambers (Xona Microfluidics LLC, CA, USA) were cleaned with 100% ethanol and glass coverslips coated overnight at 37º C with a poly-ornithine solution (0.1 mg/ml in PBS). The day after, the microfluidic chambers were bonded to glass coverslips, and the reservoirs were filled with PBS to avoid bubble formation. After an hour in the incubator, PBS was removed and matrigel was added to all the reservoirs to fully coat both neuronal and axonal compartments. Chambers were maintained in the incubator at 37°C in 5% CO_2_ for at least 1h. Immediately before seeding, matrigel was removed and NPCs were seeded in the top well of the neuronal compartment (300,000 cells/50 µl in NOMc). Chambers were then placed into the incubator for 30 min, after which the wells were topped up with NOMc. Cultures were maintained by changing media every 6 days and equilibrated every 3 days to compensate for evaporation. At DIV 40 axons crossed completely the axonal compartment[^39^](#_ENREF_39).

**Antibodies, lentiviral vectors, plasmids, and probes**

Antibodies used in this study for immunocytochemistry, immunoprecipitations, and western blots are listed in Table S1. LV-APPwt_GFP, LV-APPswe_tRFP, LV-APPwt_tRFP, and LV-EGFP_Rab5 were designed to be expressed under the human synapsin-1 promoter. Cloning and packaging were performed by Flash Therapeutics (France) and Vector Builder (VectorBuilder Inc., IL, USA). The CellLight™ Early Endosomes-RFP, BacMam 2.0 expressing Rab5 was used according to the manufacturer’s instructions (Thermo Fisher) as well as LysoTracker (LT) Deep Red (Thermo Fisher). Plasmids over-expressing APPwt, APPswe, and APPlon, were designed in a pcDNA 3.1(+) backbone, under a CMV promoter and GFP tag placed for all the three constructs in the C-terminal region of the APP. The plasmids were generated by GenScript (GenScript Biotech, New Jersey, USA), and DH5α bacterial competent cells (Thermo Fisher) were transformed in-house. Subsequently, plasmids were amplified, and stock preparations were made according to the QiaFilter Plasmid Maxi kit (Qiagen) protocol followed by DNA purification. Samples were then stored in aliquots for long-term storage at -20°C.

**Transduction, transfection, and treatments of cultured neurons and SH-SY5Y**

Human Neurons or SHSY-5Y cells were transduced with lentiviral particles at DIV 16 and DIV 9, respectively. Particles were retrieved from -80ºC and slowly thawed on ice for 20 min. Afterward, LVs were resuspended in NOMc media according to the validated M.O.I. and TU/ml provided by the manufacturer. The transduction was performed by replacing media with either NOMc or Optimem-containing lentiviral particles for approximately 24h. Solutions containing lentiviral particles were then replaced by fresh media. Transduction levels were checked every 48 h until the start of the experiments. LysoTracker Deep Red (Thermo Fisher) was used in DIV 40 human neurons previously transduced with either LV-APPwt_GFP or LV-APPswe_tRFP, for late-endosomes axonal transport imaging. Neurons were incubated with 50nM LysoTracker Deep Red for 15 min at 37°C in 5% CO_2_ after which fresh media was exchanged and cell cultures stabilized for ca. 30 min in the incubator before live imaging.

hNeurons over-expressing either APPwt, APPswe, or APPlon, were transfected in a 1:1 ratio of Lipofectamine 2000 (Thermo Fisher) and 1µg of DNA of interest. For the ibidi multichannel slide, we used 50 µl of transfection mix per channel, for a total of 300 µl of mix for all 6 channels. The transfection mix was prepared according to the manufacturer's protocol. In brief, 2 different tubes were prepared to independently incubate, at R.T. for 5 min, both Lipofectamine 2000 and the DNA of interest, in NOMc. A final transfection mix was prepared by mixing the two solutions and then incubated for 20 min at R.T.. The transfection mix was incubated with neuronal cultures for 2 h at 37º C and 5% CO_2_. Afterwards, the solution containing Lipofectamine was completely replaced by fresh NOMc, and cells were checked under an epifluorescent microscope after 24 h to monitor the fluorescent signal.

For the inhibition of the β-site APP-cleaving enzyme (BACE), either 10 µM or 40 µM of the BACE inhibitor II (Merck) were applied to DIV 20 differentiated SH-SY5Y cells. The compound was initially suspended in an appropriate volume of DMSO following the manufacturer's instructions and its solubility. Subsequently, we prepared aliquots of the BACEi at concentrations that would prevent DMSO-induced cell toxicity, achieved by diluting the compound into an Optimem complete medium. Cells were then incubated with DMSO or different concentrations of the BACE inhibitor for 2 hours at 37°C in 5% CO_2_.

**Live imaging and tracking**

Movies of axonal transport of LV-APPwt_GFP, LV-APPswe_tRFP, LV-APPwt_tRFP, LV-EGFP_Rab5, Bacmam 2.0 RFP_Rab5, LysoTracker, pcDNA3.1(+)-APPwt_GFP, pcDNA3.1(+)-APPswe_GFP, and pcDNA3.1(+)-APPlon_GFP were acquired and analysed using the same protocol. Movies were recorded at 2 fps in the case of LVs and LysoTracker, and at 4fps in the case of the plasmids, using a confocal microscope equipped with a live module (Zeiss Confocal LSM780, Zeiss Live LSM7) and an immersion oil objective 63x/1.4 NA Plan Apochromat. Time-lapse movies were processed with ImageJ before the analysis in Imaris (version 9.2, Oxford Instruments). Particles were segmented and tracked with the semi-automated spot tracking tool by applying an Autoregressive Motion algorithm. The algorithm required the input of the following parameters: XY estimated diameter, max distance, and max gap size. The diameter was chosen on an average empirical value for each specific cargo analyzed. For either max distance or max gap size, both spatial and temporal resolutions of the acquired movies were considered. Among all the statistical values obtained, those used for analyses of axonal transport parameters were the spatial displacement (∆Dx (t1, t0) = Px(t1) – Px(t2)) of the particles in each frame and the track duration (td=total time during which a particle moves). For transport dynamics analysis, we first divided the tracks into stationary or moving. All the tracks moving at < 10 s were excluded. In the net axonal transport analysis, tracks with average velocities <0.1 µm/s were defined as stationary. All the tracks with ≥0.1 µm/s velocity were considered moving and classified as anterograde or retrograde based on the sign of the average velocities: if > 0.01 µm/s = anterograde; if < - 0.01 µm/s= retrograde. In the segmental axonal transport analysis, we used the ∆X displacement between frames to compute instantaneous changes in movement, like pause frequency, reversions, segmental velocities, and real-time movement. Finally, since the software computes the distance traveled by a particle along each vector (x and y), those were shown as track lengths.

Immunocytochemistry

Neurons differentiated either in microfluidic or ibidi chambers (6 channels or 8 multi well-8mw) were fixed in 4% paraformaldehyde (PFA)/ 4% Sucrose for 1 h or 40 min, respectively. Incubation with 0.1 M Glycine for 5 min was used to quench the fixation. After that, cells were permeabilized with 0.1% Triton X-100 for 10 min. Samples were blocked with 5% BSA for 30 min and then incubated overnight at 4ºC with primary antibodies in 3% BSA. On the second day, the cells were incubated with secondary antibodies in 3% BSA for 2 h. Finally, cells were stained with DAPI for 3 min, then washed with ddH_2_O and mounted either with 50% Glycerol in 0.01% Na azide/PBS (ibidi 6 channels) or with Mowiol (ibidi 8mw and coverslips). Samples were then dried and stored at 4ºC.

**Immunohistochemistry**

Collected perfused brains were fixed in 4% PFA, embedded in the OCT Tissue Freezing Medium (Leica), and later cut into 10 µm axial sections (Leica CM1950). A mediolateral section of level 8 (2400 µm from the initially collected level) per mouse was labeled against Dynactin (p150) (1:100), APP (Y188) (1:250), MAP2 (1:500) (Table S1). Primary and secondary antibodies were diluted in antibody diluent (Dako, S302283-2). All sections were counterstained with DAPI to visualize nuclei. Antibody binding was visualized using highly cross-absorbed fluorescently labeled secondary antibodies (AlexaFluor®) (Table S1). Binding to endogenous IgG was blocked using M.O.M. blocking reagent (Vector® Laboratories, MKB-2213-1). Standard operational negative controls included sections running through the whole protocol but without incubation with the primary antibody (OPNCs). Entire brain sections were imaged on a Zeiss AxioScan.Z1 slide scanner microscope equipped with LED illumination, corresponding narrow band filter sets appropriate for each dye, and a sensitive Orca Flash 4.0 B&W camera at 10 x magnification (mounted with 1x opto-coupler). All delineations of selected regions of interest were done blinded, and measurements were done fully automated in Image Pro Premier (v9.1 or higher). The labeling segmented in total, IR surface area percentage (relative to ROI size), mean signal, mean object size, and integrated optical density were exported. Cells were counted in overlap with a minimally 20 µm^2^ DAPI nucleus to minimize 3D bias.

**Confocal imaging**

Fixed cells were examined either with an inverted Zeiss LSM 780 confocal microscope (Zeiss, Germany) or with a Leica DM 6000B (Leica Microsystems, Germany) using an oil immersion objective (63X/1.4 NA plan Apochromat). Z-stacks were acquired for the MAP2 and pNFH imaging analyses in both ibidi and microfluidic chambers. For APPwt versus APPswe localization, z-stacks in tile-scan mode were acquired to image APP distribution in whole neurons and compare their distribution in the same cell. Intensities were measured using Tau as the mask channel and distributions were represented as intensity ratios of the distal axonal regions relative to the soma. To define Rab5 particle sizes, a Lightning module (Leica microsystems) was used to deconvolve z-stacks and enhance particle resolution for an unbiased analysis of the puncta size.

**Identification of axonal projections**

Ibidi µ-Slides VI 0.4 were used to perform part of the transport experiments for both APPwt GFP and APPswe tRFP. Differently from the microfluidic chambers, the ibidi device lacks a physical division between the neuronal and axonal compartments. For this reason, prior to seeding the neurons, the ibidi were marked with an arbitrary reference point to set an X; Y (0;0), this was then matched with the 0;0 of the confocal stage before time-lapse acquisition. While acquiring movies, each selected projection was marked into a position list and saved. Post-live imaging cells were used for immunocytochemistry, following the protocol described above, and positions were retrieved to identify exclusively pNFH(+) neurites, which were included in the axonal transport analysis.

Image analysis

Images acquired following immunocytochemistry were analyzed either with ImageJ or Imaris.

(i) pNFH-GFP/tRFP post-live imaging: neurites were traced in ImageJ following GFP or tRFP intensities for APPwt_GFP and APPswe_tRFP, respectively. Intensity profiles of APP were matched with those of pNFH to either include or exclude the neurite from further axonal transport analysis.

(ii) APP intensity along projections: z-stacks mosaics were acquired such that all the projections of the neuron of interest were included in the field of view. Using Imaris software, neurons were segmented with a semi-automated approach for GFP, tRFP, and Tau (mask reference channel). Masked images were exported and further analyzed in ImageJ. Briefly, for each masked image, a maximum intensity projection was generated, and on the far-red channel (Tau) two ROIs were outlined. One included the soma of the neuron, where the mean intensity (*f_0_*) for each channel was calculated. The second ROI was traced with a line that followed the projection from the soma (0 µm) up to 200 µm away. The mean intensity (*f*) vs distance (0-200µm) was calculated for each channel, and *f/f_0_* ratios were plotted, representing normalized intensity along the projection to the respective soma. Finally, the area under the curve of *f/f_0_* was calculated for each channel in both proximal (0-100 µm) and distal (100-200 µm) regions of the projection.

(iii) Rab5 size and densities: Rab5 puncta areas were measured in deconvolved z-stacks. Tau was used as a mask. When transduced with APPswe, only those cells that showed overexpression of mutant APP were used to measure the sizes of Rab5 puncta. Masked images were analyzed in the Rab5 channel with the Analyze particles tool from ImageJ to determine the area of the puncta in the whole neuron, and their densities in distal projections.

Protein extraction

For Immunoprecipitations (IPs), proteins from 20 DIV differentiated SH-SY5Y cells, non-transduced (NT) or transduced with either APPwt GFP, APPswe_tRFP, or APPwt_tRFP as well as from hiPSCs-derived neural rosettes harvested from healthy subjects and subjects carrying APPswe were collected using the IP lysis buffer (1% Nonidet – P 40, 25 mM Tris buffer pH 7.4, 150 mM NaCl, 1mM EDTA, 5% Glycerol). Cells were incubated for 30 min on ice and cell membranes were disrupted using an insulin syringe. Supernatants were collected into new tubes following centrifugation for 20 min at 20,000xg 4ºC. Total lysates were quantified using the BCA assay according to the manufacturer's instructions (Pierce™ BCA Protein Assay Kit, Thermo Fisher).

Immunoprecipitations

Bait proteins were immunoprecipitated either with GFP- or RFP-trap Magnetic Agarose beads (Chromotek, Proteintech) or with Dynabeads Protein G (Thermo Fisher), which both allow magnetic separation of bait proteins. GFP or RFP-trap were used to IP APPwt_GFP, or APPswe_tRFP and APPwt_tRFP, respectively. Prior to the addition of proteins, beads were equilibrated with 500 µl of Dilution buffer (10 mM Tris/Cl pH 7.5, 150 mM NaCl, 0.5 mM EDTA, 0.018% Na azide) as instructed by the manufacturer’s protocol. 1 mg of total protein lysate was used per each IP. Beads were used at 50 µl/mg of total lysate for both GFP and RFP trap and samples were incubated for 2 h at 4ºC rotating end-over-end. Tubes were spun at 1000xg for 1 min and then placed into DynaMag-2 (Thermo Fisher) to separate flow-through fractions. The beads were washed twice for 2 min with 200µl/wash buffer (150 mM NaCl, 50 mM Tris/Cl pH 7.5). Protein complexes were eluted using 1X LDS sample buffer, 1X NuPage DTT reducing agent (Thermo Fisher), and heated at max 80ºC for 15 min. Eluates were transferred into new tubes to be used for SDS-PAGE and western blot analyses.

For IP of DCTN1, KLC1, and APP, 50 µl of Dynabeads Protein G were used per each sample. Beads were vortexed for 30" and freed from storing solution, then the antibody conjugation was performed by adding 200 µl of the Ab Binding Buffer (Thermo Fisher), and 5 µg of DCTN1, KLC1, or APP antibody. The antibody mix was incubated for 1 h at 4ºC rotating end-over-end. Ab-binding buffer was then discarded, and 1 mg of total lysate was added to the beads. Incubation, washes, and elution steps were performed as described above.

**SDS-PAGE and Western blotting**

Quantified protein samples were loaded into Bolt Bis-Tris Plus 4-12 % precast gels (Novex, Thermo Fisher) and SDS-PAGE was performed first for 10 min at 50 V and then at 100 V for 1 h 30 min. Proteins were then transferred onto PVDF 0.45 µm membranes (Thermo Fisher) using a Mini blot module (Invitrogen) for 1 h 50 min at 30 V. Membranes were washed 3 X 5 min with 20mM Tris-Buffer (TBS) and blocked in 5% non-fat dry milk (NFDM) in 20mM TBS/0.2% Tween-20 (TBS-T) for 1 h at RT. After 3 washes in TBS, the membranes were probed overnight at 4ºC with primary antibodies (Table S1) in 1% BSA/TBS-T 0.2%. On the second day, HRP-conjugated antibodies were prepared in 1% NFDM TBS-T 0.2% and incubated for 2 h at 4ºC. Last, the membranes were washed 3 X 5 min with TBS-T 0.2% and proteins were detected using Chemiluminescent Substrate (SuperSignal™ West Pico PLUS, Thermo Scientific) by acquiring images with Chemidoc (BioRad).

**Statistical analysis**

All the analyses were performed using GraphPad Prism 10 Statistical tests for multiple comparisons are described in detail in figure legends.

**SUPPLEMENTARY FIGURES AND LEGENDS**

**Fig. S1 – Different Familial Alzheimer's Disease mutations impair APP axonal transport.**

**A.** Experimental workflow for the transient over-expression of APPwt, APPswe, and APPlon plasmids. Neurons are transfected 48 h prior to performing live imaging, with 1 µg of DNA per each APP variant (*left*). Temporal color-coded images of APPwt, APPswe, and APPlon particles moving during an interval of 60 s recorded at 4 fps speed (*right*, scale bar = 10 µm).

**B.** Proportions (%) of APPwt, APPswe, and APPlon particles moving in the anterograde or retrograde direction, and stationary (*n* = 8 axonal projections from 3 different biological replicates).

**C.** Average velocities of anterogradely or retrogradely moving APPwt, APPswe, and APPlon particles (*n* = 10 particles per condition from 8 axonal projections and 3 different biological replicates).

**D.** Real-time movement is represented by (%) of anterograde, retrograde, and pausing particles of either APPwt, APPswe, and APPlon (*n* > 140 particles from 8 axonal projections and 3 different biological replicates).

**E.** Pauses frequency during 10 s interval quantified for APPwt, APPswe, and APPlon tracks (*n* > 140 particles from 8 axonal projections and 3 different biological replicates).

**F.** APPwt, APPswe, and APPlon reversions frequency quantified during a 10 s interval (*n* > 140 particles from 8 axonal projections and 3 different biological replicates).

Data are shown as proportions (cake plot-**B**)*,* 10-90 percentile’s box-and-whiskers **(C)**, mean ± s.e.m. (**D**), and Tukey’s box-and-whiskers **(E** and **F)**. Statistical comparisons were performed using 2-way ANOVA followed by Dunnett's **(*B*)**, Mixed-effects model followed by Tukey's **(D)** multiple comparisons test, and Kruskal-Wallis followed by Dunn's multiple comparisons test **(C**, **E,** and **F)** (**P* <0.05, ***P* <0.01, ****P* <0.001).

**Fig. S2 – Proportions of anterogradely, retrogradely, and pausing APPwt particles are not influenced by the fluorescent tag.**

**A.** Representative mosaic images of APPwt and APPswe co-expression, evidencing the low overlapping fluorescent signal for performing simultaneous APPwt and APPswe particles live imaging and subsequent axonal transport analysis (scale bar = 300 µm).

**B.** Temporal color-coded picture of APPwt_tRFP time-lapse highlighting the particles movement during all the movie intervals (0 – 30 s; scale bar = 10 µm).

**C.** Proportions of anterograde and retrograde transport or pausing APPwt_tRFP compared with APPwt_GFP particles (*n =* 15 axons from 4 different biological replicates).

Data are shown as mean ± s.e.m. Statistical analysis was performed with 2-way ANOVA followed by Šídák's multiple comparisons test.

**Fig. S3 – Axonal transport analyses performed in microfluidic chambers confirm anterograde axonal transport impairment of APPswe particles.**

**A.** Representative micrographs of neurons cultured in microfluidic chambers showing pNFH (white) and MAP2 (magenta) staining (scale bar = 300 µm).

**B.** Proportions (%) of anterograde, retrograde, and stationary particle populations from neurons over-expressing either APPwt or APPswe, analyzed from axonal projections imaged in microfluidic chambers (*n* > 4 axons from 3 biological replicates).

Data are shown as mean ± s.e.m. Statistical analysis was performed with 2-way ANOVA followed by Šídák's multiple comparisons test (*** *P* < 0.001).

**Fig. S4 – Comparable distribution of APPwt immunoreactivity in the axonal projections of human neurons transduced with either tRFP or GFP.**

**A.** Representative image of a neuronal culture co-transduced with APPwt GFP and APPwt tRFP, followed by immunofluorescence against GFP, tRFP, and tau (*left*, scale bar = 100µm). Representative image of a segmented neuron before analysis of the 3 independent markers (GFP, tRFP, and tau) (*right*).

**B.** Segmentation images of the neuron in (*A*) each corresponding to an independent channel: APPwt GFP, APPwt tRFP, and tau (scale bar = 100 µm).

**C.** Graph showing the intensities (*f*) of APPwt GFP, APPwt tRFP, and tau signal along each point of the axon (0-200 µm) relativized to their respective mean intensity in the soma (*f_0_*) (*n* = 20 neurons from 6 biological replicates).

**D.** Quantification of the total intensity (A.U.C.= area under the curve) of APPwt GFP and APPwt tRFP along the axon in the first 100 µm from the soma (0-100 µm), or further away (100-200 µm) (*n* = 20 neurons from 6 biological replicates).

Data are shown as dots representing the mean intensity values at distance *x* from the soma, fitted and smoothed by a continuous line **(C)**, and A.U.C. values with mean bar plot and independent values **(D)**. Statistical comparison was performed using a 2-way ANOVA (matched by biological replica) followed by Šídák's multiple comparisons test **(D)**.

**Fig. S5 – Immunoblots of APPwt, APPswe, and non-transduced samples, following IPs, and quantification of APP, DCTN1, and KLC1 levels in total lysates fractions.**

**A.** Uncropped membranes of IP samples obtained from GFP and tRFP immunoprecipitations (IPs) prepared with cell lysates transduced with either APPwt or APPswe, followed by immunoblots against APP, DCTN1, KLC1, and βIII-tubulin (loading control). Intensities are shown for both input (10 µg of protein per lane), and IP samples, where DCTN1, and KLC1 co-IP bands are part of either APPwt or APPswe immunocomplexes. The full-membrane western blot images correspond to the cropped key bands shown in Fig.4B. The protein ladder was always imaged soon after the exposition of the bands of interest, and then the images merged for the proper marker-molecular weight correlation.

**B.** Densitometric analysis (A.U. = arbitrary units) of APP, DCTN1, and KLC1 levels in input normalized to βIII-tubulin (loading control) (*n* > 6 biological replicates).

**C.** Control immunoblot of APPwt IP samples using GFP antibody, exclusively recognizing the APPwt _GFP over-expressed protein.

**D.** Control immunoblot of APPswe IP samples using GFP antibody, exclusively recognizing the APPswe _tRFP over-expressed protein.

**E.** Representative immunoblot of APP from non-transduced (NT) control samples following IP against GFP, and tRFP, showing absence of any non-specific signal.

Data are represented as mean ± s.e.m and statistical comparison was performed using a 2-way ANOVA followed by Šídák's multiple comparisons tests **(B)**.

**Fig. S6** **– Immunoprecipitations of APPwt and APPswe both coupled to the tRFP tag show enhanced recruitment of DCTN1, but not KLC1, to the APP motor assemblies.**

**A.** Schematic of the co-IP experimental approach to identify interactions between APP and motor proteins machinery.

**B.** Representative immunoblots of APP, DCTN1, KLC1, and βIII-tubulin (loading control) were performed with samples obtained from tRFP IPs prepared with cell lysates transduced with either APPwt or APPswe.

**C.** Co-IP relative (to APP) intensity ratios of DCTN1 and KLC1 quantified in both APPwt and APPswe samples (*left*, *n* = 6 biological replicates). Densitometric analysis of immunoprecipitated levels of APP normalized by its corresponding input (10µg of proteins per lane) from both APPwt and APPswe transduced cell cultures (*right*, *n* =6 biological replicates).

**D.** Densitometric analysis (A.U. = arbitrary units) of APP, DCTN1, and KLC1 input normalized to β-actin (loading control) (*n* = 6 biological replicates).

**E.** Immunoblot image of tRFP antibody recognizing exclusively over-expressed APPwt and APPswe protein bands.

**F.** Representative immunoblot of APP from non-transduced (NT) control samples following IP against tRFP and showing the absence of any non-specific signal.

Data are shown as mean ± s.e.m **(B** and **C)**. Statistical comparisons were performed using an unpaired t-test **(B** left**)**, one-sample *t*-test (theoretical mean = 1) **(B** right**)**, and 2-way ANOVA followed by Šídák's multiple comparisons test **(C)** (**P* < 0.05).

**Fig. S7 – DCTN1 or KLC1 immunocomplexes analyses reveal no changes in the input protein levels of DCTN1, KLC1, and APP.**

**A.** Uncropped membranes showing immunoblots of DCTN1, APP, and βIII-tubulin (loading control). Intensities are shown for both input (10 µg of protein per lane), and IP samples, where APP co-IP bands are part of the DCTN1 immunocomplex. The full-membrane western blot images correspond to the cropped key bands shown in Fig.4C. The uncropped membrane blot for APP intensities was partially covered during acquisition to avoid overblown bands from appearing in the image, due to co-elution of heavy and light chain bands to the antibody used in the experiment from the bead-antibody complex.

The protein ladder was always imaged soon after the exposition of the bands of interest, and then the images merged for the proper marker-molecular weight correlation.

**B.** Densitometric analysis (A.U. = arbitrary units) of APP and DCTN1 levels in input normalized to βIII-tubulin (loading control) (*n* = 4 biological replicates).

**C.** Uncropped membranes showing immunoblots of KLC1, APP, and βIII-tubulin (loading control). Intensities are shown for both input (10 µg of protein per lane), and IP samples, where APP co-IP bands are part of the KLC1 immunocomplex. The full-membrane western blot images correspond to the cropped key bands shown in Fig.4D. The uncropped membrane blot for APP intensities was partially covered during acquisition to avoid overblown bands from appearing in the image, due to co-elution of heavy and light chain bands of the antibody used in the experiment from the bead-antibody complex.

The protein ladder was always imaged soon after the exposition of the bands of interest, and then the images merged for the proper marker-molecular weight correlation.

**D.** Densitometric analysis (A.U. = arbitrary units) of APP and KLC1 levels in input normalized to βIII-tubulin (loading control) (*n* = 3 biological replicates).

Data are represented as mean ± s.e.m, while statistical comparison was performed using a 2-way ANOVA followed by Šídák's multiple comparisons test **(B** and **D)**.

**Fig. S8 – Immunoblots from hiPSCs-derived neural progenitors from healthy control and AD APPswe patient.**

**A.** Immunoblots against DCTN1, APP, and β-actin following IP against either APP or IgG isotype control. The full-membrane western blot images correspond to the cropped key bands shown in Fig.4E. The protein ladder was always imaged soon after the exposition of the bands of interest, and then the images merged for the proper marker-molecular weight correlation.

**B.** Densitometric analysis (A.U. = arbitrary units) of APP and DCTN1 input normalized to β-actin (loading control) (*n* = 3 biological replicates).

Data are represented as mean ± s.e.m, while statistical comparison was performed with 2-way ANOVA followed by Šídák's multiple comparisons test.

**Fig. S9 – IPs of SH-SY5Y over-expressing APPswe tRFP following BACEi treatment.**

**A.** Schematic of the APP proteolytic processing and location of the two main FAD mutations examined in the current study, APPswe (red star) and APPlon (blue star).

**B.** Immunoblots representing DCTN1, APP, and β-actin intensities following IP against tRFP after treatments with DMSO, BACEi 10µm, or BACEi 40 µm.

**C.** IP efficiency of APP (*left* graph) and DCTN1(*right* graph) levels normalized by their corresponding input (10 µg of proteins per lane) from either APPswe transduced cell cultures following DMSO, BACEi 10µm, or BACEi 40µm treatments (*n* = 12 biological replicates).

**D.** Co-IP ratios expressed as DCTN1 intensity relative to either APPswe transduced cell cultures following DMSO, BACEi 10µm, or BACEi 40µm treatments (*n* = 12 biological replicates).

**Fig. S10** **– hNSCs-derived neurons over-expressing APPwt do not show changes in axonal transport of Rab5.**

**A.** Real-time movement (%) of anterograde, retrograde, or pausing Rab5+ particles in Rab5 or Rab5+APPwt transduced cell cultures (*n* > 50 particles from 3 biological replicates).

**B.** Track lengths (distances) of Rab5+ trajectories in Rab5 or Rab5+APPwt transduced cell cultures (*n* > 50 particles from 3 biological replicates).

**C.** 10 s interval pauses frequencies of Rab5+ particles in Rab5 or Rab5+APPwt transduced cell cultures (*n* > 50 particles from 3 biological replicates).

**D.** 10 s interval reversion frequencies of Rab5+ particles analyzed in Rab5 or Rab5+APPwt transduced cell cultures (*n* > 50 particles from 3 biological replicates).

**E.** Velocities of anterogradely (*left*) and retrogradely (*right*) transported Rab5+ particles quantified either in Rab5 or Rab5+APPwt transduced cell cultures. Velocities >0.1 µm/s are represented as cumulative frequency distributions. Subgraphs represent box-and-whiskers distributions of anterograde and retrograde populations in Rab5 and Rab5+APPwt transduced neurons (*n* > 750 segments from more than 50 trajectories analyzed from 3 biological replicates).

**F.** Representative immunoblots of Rab5 and βIII-tubulin (loading control) levels in controls non-transduced (*ctrl* NT), and APPwt or APPswe transduced SH-SY5Y differentiated cell cultures.

**G.** Densitometric analysis of Rab5 levels normalized to βIII-tubulin (loading control), in *ctrl* NT, and APPwt or APPswe transduced SH-SY5Y differentiated cell cultures.

Data are represented as mean ± s.e.m. (**A** and **G**), Tukey’s box-and-whisker plot (**B**-**D,** and subgraphs in **E**), and cumulative frequency distributions (**E**). Statistical comparisons were performed using 2-way ANOVA followed by Šídák's multiple comparisons test (**A**), Mann-Whitney *U* test (**B**-**E**), and One-way ANOVA followed by Tukey's multiple comparisons test **(G)** (**P* < 0.05, ***P* < 0.01).

**Table S1 - Antibodies used in the study.**

**SUPPLEMENTARY MOVIES LEGENDS**

**Movie 1 – Transport in the projections of APPwt_GFP transfected human neurons.**

Video of APPwt particles recorded using a 489 nm laser at 4 fps: directionality of movement left (proximal to the cell body) to right (distal from the cell body). Scale bar = 10 µm.

**Movie 2 – Transport in the projections of APPswe_GFP transfected human neurons.**

Video of APPswe particles recorded using a 489 nm laser at 4 fps: directionality of movement left (proximal to the cell body) to right (distal from the cell body). Scale bar = 10 µm.

**Movie 3 – Transport in the projections of APPlon_GFP transfected human neurons.**

Video of APPlon particles recorded using a 489 nm laser at 4 fps: directionality of movement left (proximal to the cell body) to right (distal from the cell body). Scale bar = 10 µm.

**Movie 4 – Axonal transport of APPwt_GFP transduced in human neurons.**

Video of APPwt particles recorded using a 489 nm laser at 2 fps: directionality of movement left (proximal to the cell body) to right (distal from the cell body). Scale bar = 10 µm.

**Movie 5 – Axonal transport of APPswe_tRFP transduced in human neurons.**

Video of APPswe particles recorded using a 561 nm laser at 2 fps: directionality of movement left (proximal to the cell body) to right (distal from the cell body). Scale bar = 10 µm.

**Movie 6 – Semi-automated segmentation and tracking of APPwt_GFP particles generated using Imaris software.**

Video of APPwt particles segmentation and tracking using IMARIS spots track analysis. Recordings were made using a 489 nm laser at 2 fps: directionality of movement left (proximal to the cell body) to right (distal from the cell body). Scale bar = 10µm, time scale bar = 0-30 seconds.

**Movie 7 – Semi-automated segmentation and tracking of APPswe_tRFP particles generated using Imaris software.**

Video of APPswe particles segmentation and tracking using IMARIS spots track analysis. Recordings were made using a 489 nm laser at 2 fps: directionality of movement left (proximal to the cell body) to right (distal from the cell body). Scale bar = 10µm, time scale bar = 0-30 seconds.

**Movie 8 – Axonal transport of APPwt_tRFP transduced in human neurons.**

Video of APPwt particles recorded using a 561 nm laser at 2 fps: directionality of movement left (proximal to the cell body) to right (distal from the cell body). Scale bar = 10 µm.

**Movie 9 – Axonal transport of RFP_Rab5 in human neurons co-transduced with APPwt_GFP.**

Video of Rab5 particles recorded using a 561 nm laser at 2 fps: directionality of movement left (proximal to the cell body) to right (distal from the cell body). Scale bar = 10 µm.

**Movie 10 – Axonal transport of EGFP_Rab5 in human neurons co-transduced with APPswe_tRFP.**

Video of Rab5 particles recorded using a 489 nm laser at 2 fps: directionality of movement left (proximal to the cell body) to right (distal from the cell body). Scale bar = 10 µm.

**Movie 11 – Axonal transport of LysoTracker DeepRed in human neurons co-transduced with APPwt GFP.**

Video of Rab5 particles recorded using a 633 nm laser at 2 fps: directionality of movement left (proximal to the cell body) to right (distal from the cell body). Scale bar = 10 µm.

**Movie 12 – Axonal transport of LysoTracker DeepRed in human neurons co-transduced with APPswe tRFP.**

Video of Rab5 particles recorded using a 633 nm laser at 2 fps: directionality of movement left (proximal to the cell body) to right (distal from the cell body). Scale bar=10 µm.
